# High crude violacein production from glucose by *Escherichia coli* engineered with interactive control of tryptophan pathway and violacein biosynthetic pathway

**DOI:** 10.1186/s12934-015-0192-x

**Published:** 2015-01-16

**Authors:** Ming-Yue Fang, Chong Zhang, Song Yang, Jin-Yu Cui, Pei-Xia Jiang, Kai Lou, Masaaki Wachi, Xin-Hui Xing

**Affiliations:** Department of Chemical Engineering, Tsinghua University, Beijing, 10084 China; School of Life Sciences, Qingdao Agriculture University, Qingdao, 266109 China; Institute of Microbiology, Chinese Academy of Science, Beijing, 10084 China; Institute of Microbiology, Xinjiang Academy of Agricultural Science, Urumqi, 830000 China; Department of Bioengineering, Tokyo Institute of Technology, Yokohama, 226-8503 Japan

**Keywords:** Violacein, Tryptophan, Glucose, Pathway optimization, *Escherichia coli*

## Abstract

**Background:**

As bacteria-originated crude violacein, a natural indolocarbazole product, consists of violacein and deoxyviolacein, and can potentially be a new type of natural antibiotics, the reconstruction of an effective metabolic pathway for crude violacein (violacein and deoxyviolacein mixture) synthesis directly from glucose in *Escherichia coli* was of importance for developing industrial production process.

**Results:**

Strains with a multivariate module for varied tryptophan productivities were firstly generated by combinatorial knockout of *trpR/tnaA/pheA* genes and overexpression of two key genes *trpE*^*fbr*^*/trpD* from the upstream tryptophan metabolic pathway. Then, the gene cluster of violacein biosynthetic pathway was introduced downstream of the generated tryptophan pathway. After combination of these two pathways, maximum crude violacein production directly from glucose by *E. coli* B2/pED + pVio was realized with a titer of 0.6 ± 0.01 g L^−1^ in flask culture, which was four fold higher than that of the control without the tryptophan pathway up-regulation. In a 5-L bioreactor batch fermentation with glucose as the carbon source, the recombinant *E. coli* B2/pED + pVio exhibited a crude violacein titer of 1.75 g L^−1^ and a productivity of 36 mg L^−1^ h^−1^, which was the highest titer and productivity reported so far under the similar culture conditions without tryptophan addition.

**Conclusion:**

Metabolic pathway analysis using ^13^C labeling illustrated that the up-regulated tryptophan supply enhanced tryptophan metabolism from glucose, whereas the introduction of violacein pathway drew more carbon flux from glucose to tryptophan, thereby contributing to the effective production of crude violacein in the engineered *E. coli* cell factory.

**Electronic supplementary material:**

The online version of this article (doi:10.1186/s12934-015-0192-x) contains supplementary material, which is available to authorized users.

## Introduction

Violacein is a natural indolocarbazole product obtained from bacteria, which could potentially be a new type of natural antibiotic [1] owing to its promising bioactivities such as antibacterial [2], antitumoral [3], antiviral [4], trypanocidal [5], antiprotozoan [6], and antioxidant activities [7]. Since both violacein and deoxyviolacein have the similar acid/alkali resistance, UV resistance and antibacterial activity [8], the mass production of crude violacein (mixture of violacein and deoxyviolacein) with an adequate quantity at low cost is of significant importance.

In previous studies, *Chromobacterium violaceum, Duganella* sp., and *Janthinobacterium lividum* were isolated for violacein production, which exhibited violacein titers of 1.1, 1.62, and 0.85 g L^−1^, respectively [3,9,10]. However, the wild violacein producers were found to form non-violacein-producing variants at a high frequency during culture and subculture, which was one of the key limiting factors for their industrial application [6], also, *C. violaceum* and *J. lividum* were also find to be potential pathogens which limit their industrial application [11,12]. Therefore, reconstruction of violacein biosynthetic pathway in industrially acceptable hosts for stable and high production of violacein in either crude or pure form has been attracting increasing attention.

*Citrobacter freundii* and *Enterobacter aerogenes* [6] had been employed as hosts for the reconstruction of violacein biosynthetic pathway in our group. Among them, *C. freundii* could achieve the highest titer of 4.12 g L^−1^ crude violacein with the addition of 2.8 g L^−1^ tryptophan in fed-batch fermentation [13]. However, owing to the high cost of tryptophan (an essential amino acid), engineering of strains for violacein production from cheaper carbon sources is desirable from an economic viewpoint. Furthermore, *C. freundii* is a rare but infective bacterial strain and its genome has not been sequenced, thus leading to the difficulties in genetic optimization. Therefore, a completely genetically defined and industrially acceptable host could provide more opportunities for pathway engineering. Recently, Rodrigues et al. used systems metabolic engineering to successfully reconstruct a violacein production pathway in *Escherichia coli*, and achieved a pure violacein titer of 0.71 g L^−1^ from arabinose [14] and a pure deoxyviolacein titer of 1.6 g L^−1^ from glycerol [15] in a fed-batch fermentation without the addition of tryptophan. However, the violacein and deoxyviolacein productivity of the engineered *E. coli* by the direct utilization of sugar or glycerol is still low (2 and 8 mg L^−1^ h^−1^, respectively) [14,15], when compared with that achieved from tryptophan of external addition using other recombinant strains such as *C. freundii* [13]. Moreover, the use of arabinose as the carbon source for the recombinant *E. coli* is unsuitable for industrial application from an economic viewpoint. To the best of our knowledge, no successful trials have been conducted to reconstruct the violacein biosynthetic pathway in *E. coli* for crude or pure violacein production directly from glucose without the addition of tryptophan.

Violacein biosynthetic pathway was first studied by Pemberton et al. [16] and was completely elucidated by Balibar et al. [17] and Sanchez et al. [18] in 2006. Tryptophan, as a substrate for the synthesis of violacein, is catalyzed by VioA to form indole-3-pyruvic acid, which is then converted to protodeoxyviolacein acid by VioB and VioE, followed by the catalysis of VioD and VioC to form violacein and deoxyviolacein as the dominant product and byproduct, respectively. With regard to the metabolic engineering of a long pathway with indigenous precursor supply and target metabolite synthesis, the whole pathway can always be separated into several modules for further optimization [19], which will facilitate the design and control of the interactive pathways to achieve high titer of the target metabolite [20,21]. For the construction of efficient violacein biosynthetic pathway from glucose, tryptophan could be the key node for separating the upstream and downstream pathways. Previous studies have found that the addition of tryptophan to the medium can increase violacein production by both violacein-producing wild strains and recombinant strains [13,22]. In addition, Rodrigues et al. [14] also demonstrated that an increase in the supply of tryptophan through pathway engineering could benefit violacein production from arabinose by *E. coli*. However, to date, the way in which the violacein biosynthetic pathway could affect the upstream tryptophan pathway remains unknown. Thus, a modular approach with tryptophan as the key node for the violacein biosynthetic pathway could be a useful metabolic engineering strategy to achieve high violacein production by *E. coli* from glucose as the carbon source.

In the present study, deregulated *trpE*^*fbr*^ and *trpD* genes were introduced into *E. coli* mutants with combinatorial knockout of gene(s) *trpR/pheA/tnaA* to generate strains with different tryptophan productivities. Subsequently, the *vio* gene cluster (*vioABCDE*) for violacein biosynthetic pathway was introduced into the engineered strains with multivariate tryptophan pathway to examine their combinatorial effects on violacein production from glucose. Then, the optimum strain with the highest violacein titer was chosen for further analysis. Metabolic pathway analysis using ^13^C labeling was performed to elucidate the carbon flux change in the pathway network. The tryptophan yield was also estimated to uncover the pulling of the upstream pathway (tryptophan pathway) by the downstream pathway (violacein pathway). Subsequently, batch fermentation of the optimized recombinant strain in a 5-L bioreactor was performed using glucose as the carbon source to demonstrate the potential of the high violacein producer.

## Results

### Generation of strains with multivariate tryptophan pathway module

**Figure 1:**
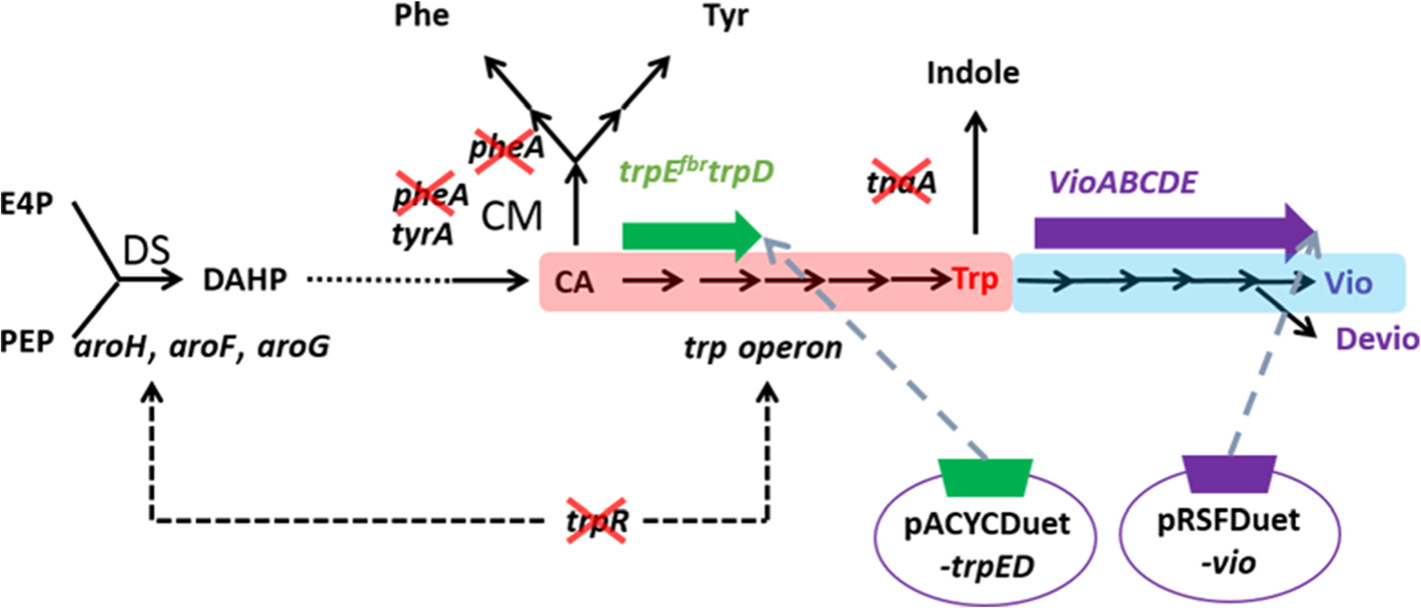
**Plasmid construction and gene knockout related to tryptophan accumulation.** The black dotted line indicates repression. The red cross indicates the target genes to be knocked out. The pink and blue colors indicate the upstream pathway of tryptophan accumulation, which was improved, and the downstream pathway of violacein production from tryptophan, which was introduced, respectively. E4P, erythrose 4-phosphate; PEP, phosphoenolpyruvate; DS, 3-deoxy-D-arabinoheptulosonate 7-phosphate synthase; DAHP, 3-deoxy-D-arabinoheptulosonate 7-phosphate; CM, chorismate mutase; CA, chorismic acid; Phe, phenylalanine; Tyr, tyrosine; Trp, tryptophan; Vio, violacein, Devio, deoxyviolacein.

The tryptophan biosynthetic pathway has been well studied in *E. coli* and *Corynebacterium glutamicum*. The pathway starts with the condensation of phosphoenolpyruvate (PEP) and erythrose 4-phosphate (E4P), and then forms chorismate, followed by the tryptophan branch pathway to form tryptophan. In addition, phenylalanine and tyrosine branches also exist, which compete for chorismate to form phenylalanine and tyrosine [23]. The metabolic flow of tryptophan pathway is always controlled by tryptophan feedback inhibition, attenuation, and repression (Figure 1) [23]. Thus, it is possible to generate tryptophan-accumulating strains by rational design such as 1) enhancing the activity of key enzymes in the common pathway to form chorismate and tryptophan operon to improve pathway metabolic flux and 2) deleting genes from chromosome that are responsible for the inhibition of tryptophan accumulation by the repression of transcription of tryptophan operon (*trpR*), competition of chorismate to form other aromatic amino acid (*pheA*), and degradation of tryptophan (*tnaA*) [23-25].

**Figure 2:**
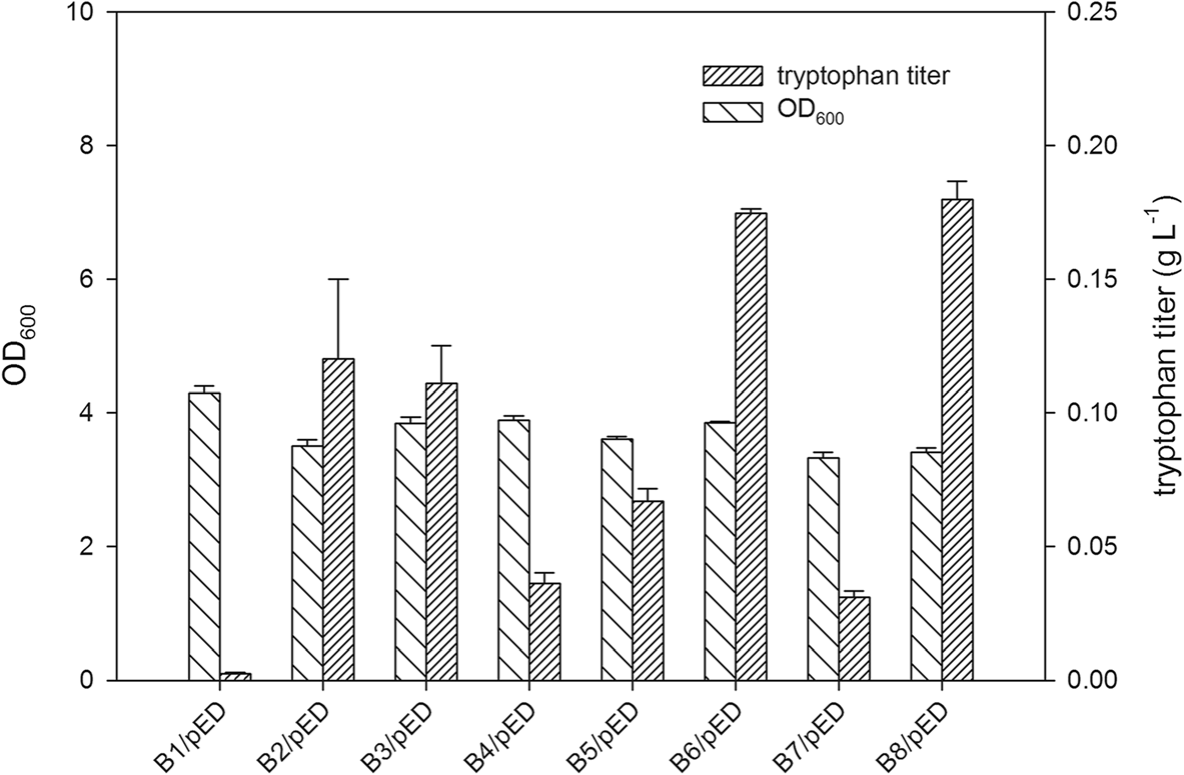
**Production of tryptophan in flask culture with M9-YE medium (10 g L** ^**−1**^ **glucose) at 20°C for 48 h (n=3).**

The multivariate module for various tryptophan-producing strains were generated by combining the overexpression of *trpE*^*fbr*^ (feedback inhibition removed by point mutation Met293Thr and Ser40Leu) and *trpD* under T7 promoter, which are reported to be the key genes to enhance tryptophan synthesis [23] and combinatorial knockout of *trpR, tnaA,* and *pheA*, generating eight different BL21(DE3) strains, B1–B8, possibly having different capabilities of producing tryptophan (Table 1). The eight engineered strains were subsequently cultivated in flasks with optimized culture medium (Additional file 1: Table S2, Table S3 and Table S4) for tryptophan accumulation, as shown in Figure 2. B6/pED and B8/pED showed the highest tryptophan concentration (around 0.18 g L^−1^), while B2/pED and B3/pED were the second highest tryptophan-producing strains, and B1/pED were the lowest tryptophan producers.

**Table 1:** **Strains and plasmids used in this study**

**Strains or plasmids**	**Characteristics**	**Source**
**Strains**		
**B1**	*E. coli* BL21(DE3) wild type	Laboratory stock
**B2**	*E. coli* BL21(DE3) *ΔtrpR*	This study
**B3**	*E. coli* BL21(DE3) *ΔtrpR ΔpheA*	This study
**B4**	*E. coli* BL21(DE3) *ΔtnaA*	This study
**B5**	*E. coli* BL21(DE3) *ΔtnaA ΔpheA*	This study
**B6**	*E. coli* BL21(DE3) *ΔtrpR ΔtnaA*	This study
**B7**	*E. coli* BL21(DE3) *ΔpheA*	This study
**B8**	*E. coli* BL21(DE3) *ΔtrpR ΔtnaA ΔpheA*	This study
**plasmids**		
**pACYCDuet**	*Cm*	Novagen
**pRSFDuet**	*Km*	Novagen
**pED**	pACYCDuet with *trpE* ^*fbr*^ (Met293Thr, TCC to TTC; Ser40Leu ATG to ACG) and *trpD* genes in NcoI and KpnI sites	This study
**pVio**	pRSFduet with *vio* gene cluster in NdeI and XhoI sites	This study

### Combination of upstream and downstream pathways for violacein production

**Figure 3:**
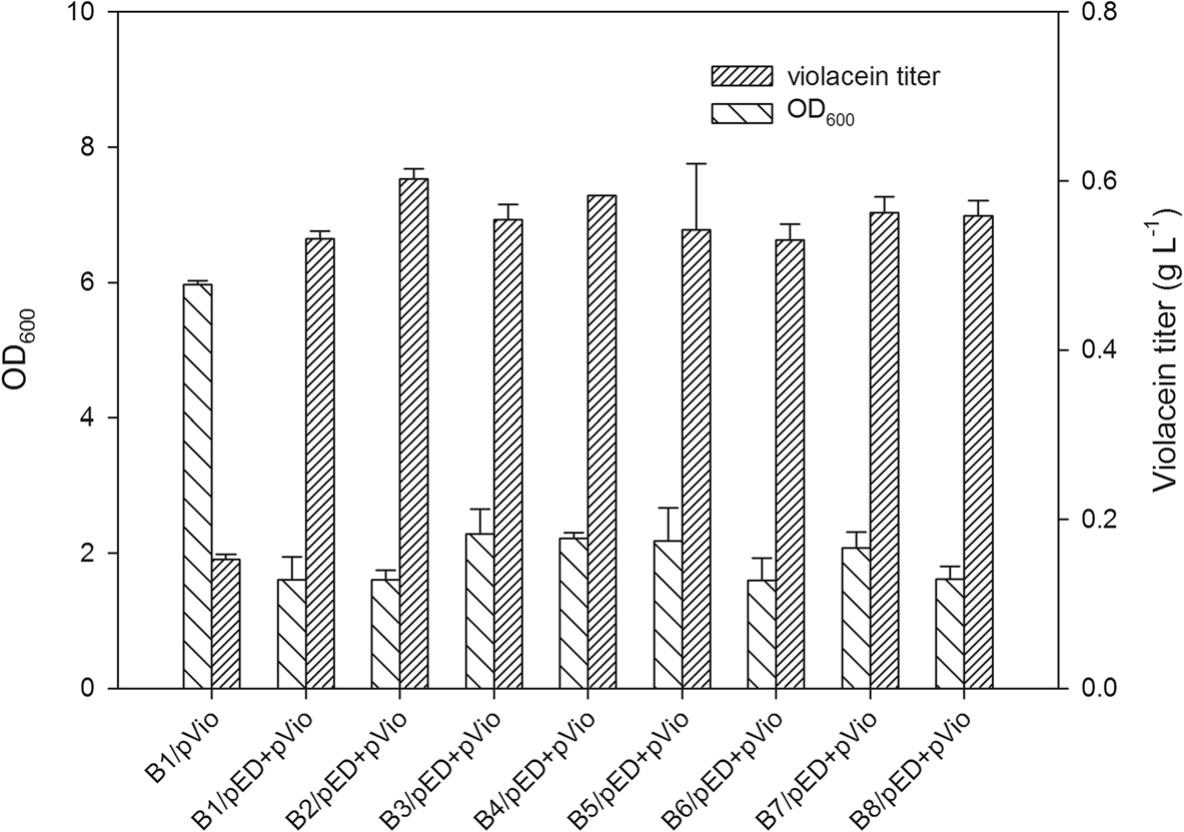
**Production of crude violacein in flask culture with M9-YE medium (10 g L** ^**−1**^ **glucose) at 20°C for 48 h (n=3).**

**Figure 4:**
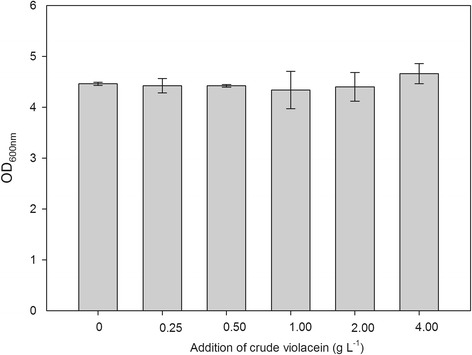
**The tolerance of** ***E. coli*** **Bl21(DE3) to exogenously added crude violacein (violacein and deoxyviolacein mixture) (n=3).**

The plasmids pACYCDuet-*trpE*^*fbr*^*/trpD*(pED) and pRSFDuet-*vio* (pVio) were co-transformed into the strains B1–B8, generating eight engineered strains, while the B1 strain transformed only with pVio was used as the control. These nine strains were cultivated in 100-mL flasks containing the optimized medium (Additional file 1: Table S2, Table S3 and Table S4) for violacein production. After the flask culture, the crude violacein concentration and OD_600_ value were measured. From Figure 3, it can be noted that 1) when compared with the control strain containing only pVio, the strains harboring the two plasmids, which contained the upstream and downstream pathways exhibited 3–4-fold higher violacein titers, and 2) strains of B1-B8 which contained two plasmids (pED and pVio) shared similar violacein titers and the titers did not correlate well to the order of tryptophan production by the strains containing only one plasmid (pED) (Figure 3 vs. Figure 2), suggesting that the tryptophan pathway of poor-tryptophan producers had the possibilities to be activated to supply enough precursor when the downstream pathway for violacein synthesis was docked. The crude violacein produced by B2/pED + pVio reached 0.603 ± 0.01 g L^−1^, which was 3.94-fold higher than that produced by the control strain B1/pVio (0.153 ± 0.005 g L^−1^) (Figure 3). The OD_600_ value indicated that B1/pVio showed higher cell density than B1/pED + pVio, B2/pED + pVio and other strains with the two plasmids; however, the strain with higher cell density did not produce higher violacein concentration. Additional file 1: Table S5 also showed the fermentation result of the B1 strain with the empty plasmid pACYCDuet and pVio, its performance was similar with B1/pVio, indicating that the lower biomass of strains with the two plasmids was not caused by the addition of antibiotics. Moreover, the tolerance of *E. coli* Bl21(DE3) to exogenously added crude violacein showed that even the crude violacein concentration was as high as 4.0 g L^−1^, the growth of Bl21(DE3) was not influenced (Figure 4), which was consistence with previous report [8] that violacein and deoxyviolacein did not inhibit cell growth of *E. coli*. It suggested that the lower cell densities of B1/ pED + pVio and B2/pED + pVio were not caused by the toxicity of crude violacein produced.

### Analysis of the ^13^C incorporation rate of the metabolites in central metabolism and tryptophan pathway of B2/pED + pVio and B1/pVio

**Figure 5:**
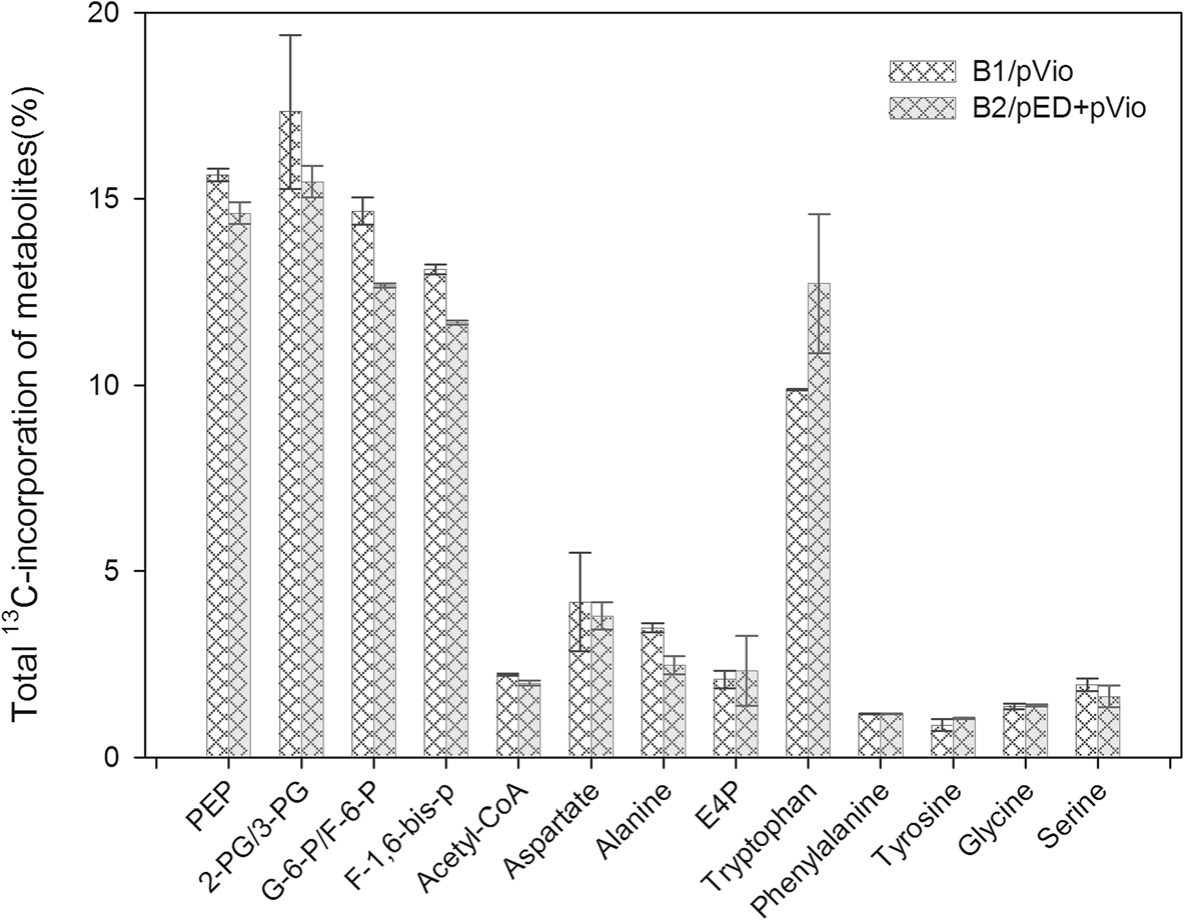
**The** ^**13**^ **C incorporation rates of different metabolites for B2/pED+pVio and B1/pVio at 20 min.** G-6-P/F-6-P, fructose-6-phosphate/glucose-6-phosphate; F-1,6-bis-P, fructose-1,6-bisphosphate; 2-PG/3-PG, 2-phosphoglycerate/3-phosphoglycerate; PEP, phosphoenolpyruvate; E4P, erythrose 4-phosphate (n=3).

To explain the differences in the phenotypes of B2/pED + pVio and B1/pVio (control), the dynamic ^13^C incorporation rate of the intermediates was monitored through a ^13^C-tracing experiment (D-Glucose-6-^13^C was initially used as the carbon source). The time course of the ^13^C incorporation rate of the intermediates was first determined to find a relatively steady state, i.e. the middle exponential phase, where the pool size is thought to be stable, the ^13^C incorporation rate of a metabolite only depends on its flux, and the higher incorporation rate means higher flux goes through that intermediates [26]. It was found that after 20 min of culture in ^13^C plate, the total ^13^C incorporation rates of most of the intermediates were nearly steady (Additional file 1: Figure S7A); thus, a culture period of 20 min was chosen for the subsequent analysis. As shown in Figure 5, the total ^13^C incorporation rates of the intermediates involved in the glycolysis pathway, such as G-6-P/F-6-P (fructose-6-phosphate/glucose-6-phosphate), F-1,6-bis-P (fructose-1,6-bisphosphate), 2-PG/3-PG (2-phosphoglycerate/3-phosphoglycerate) and PEP (phosphoenolpyruvate), were relatively lower for B2*/*pED + pVio, when compared with those for the control strain B1/pVio, indicating the lower glucose uptake rate of B2*/*pED + pVio. As to the fermentation in 20 ml flask, the real glucose uptake rate of B1/pVio (3.25 mg h^−1^) was higher than that of B2/pED + pVio (1.21 mg h^−1^) which confirmed the result of the ^13^C incorporation rate. The intermediates involved in the TCA cycle and the related intermediates such as acetyl-CoA and aspartate shared similar ^13^C incorporation rates in both strains. The pattern was also observed for serine and E4P, which were precursors for tryptophan synthesis. However, the other two important precursors, chorismate and shikimate, were not detected by LC-MS or GC-MS even with a modified extraction method (data not show). With regard to the long pathway from glucose to tryptophan, as an important precursor for violacein synthesis, another ^13^C-tracing experiment was employed using D-glucose-^13^C_6_ as the carbon source to focus on the ^13^C incorporation rate of tryptophan. The results obtained suggested that the metabolic rate of tryptophan observed for B2/pED + pVio was higher than that for B1/pVio (Figure 5). In addition, the labeling pattern of other metabolites, including glycine, phenylalanine, and tyrosine in the branch pathway was similar between B2/pED + pVio and B1/pVio.

### Change in the tryptophan and tryptophan equivalent yields of the strains after introduction of violacein biosynthetic pathway

The total tryptophan yield of four strains B1/pED, B2/pED, B1/pED + pVio, and B2/pED + pVio were determined to further explore the change in the carbon flux after reconstruction of violacein pathway. The four strains were cultivated in flasks under the same conditions at 20°C for 48 h for tryptophan or violacein synthesis. Since no tryptophan was detected in the strain of B1/pED + pVio and B2/pED + pVio, the crude violacein production of them was converted into tryptophan equivalents, as described in the Material and Methods Section. As shown in Table 2, after the reconstruction of violacein pathway, the tryptophan yields of both B1/pED + pVio and B2/pED + pVio were significantly increased when compared with those of B1/pED and B2/pED (0.13 vs. 0.0004 and 0.38 vs. 0.024, a 300- and 15-fold dramatic increase in tryptophan equivalent yield, respectively), indicating the powerful pulling effect of the downstream violacein biosynthesis pathway on the upstream precursor supply.

**Table 2:** **C-mol of biomass, tryptophan, and tryptophan equivalent yields from glucose of different strains (n=3)**

**Strains**	**Glc consumption (g L** ^**−1**^ **)**	**Actual tryptophan titer (g L** ^**−1**^ **)**	**Equal tryptophan titer (g L** ^**−1**^ **)**	**OD**_**600**_	**C-mol biomass yield**	**C-mol tryptophan yield**
**B1/pED**	8.2±0.2	0.0024±0.0005	-	4.3±0.1	0.20±0.01	0.0004±0.0001
**B2/pED**	8.0±0.2	0.12±0.03	-	3.5±0.1	0.16±0.01	0.024±0.005
**B1/pED+pVio**	7.6±0.3	-	0.60±0.01	1.6±0.4	0.078±0.03	0.13±0.01
**B2/pED+pVio**	2.9±0.3	-	0.68±0.01	1.6±0.3	0.21±0.03	0.38±0.01

### Fermentation kinetics of B2/pED + pVio in 5-L bioreactor

**Figure 6:**
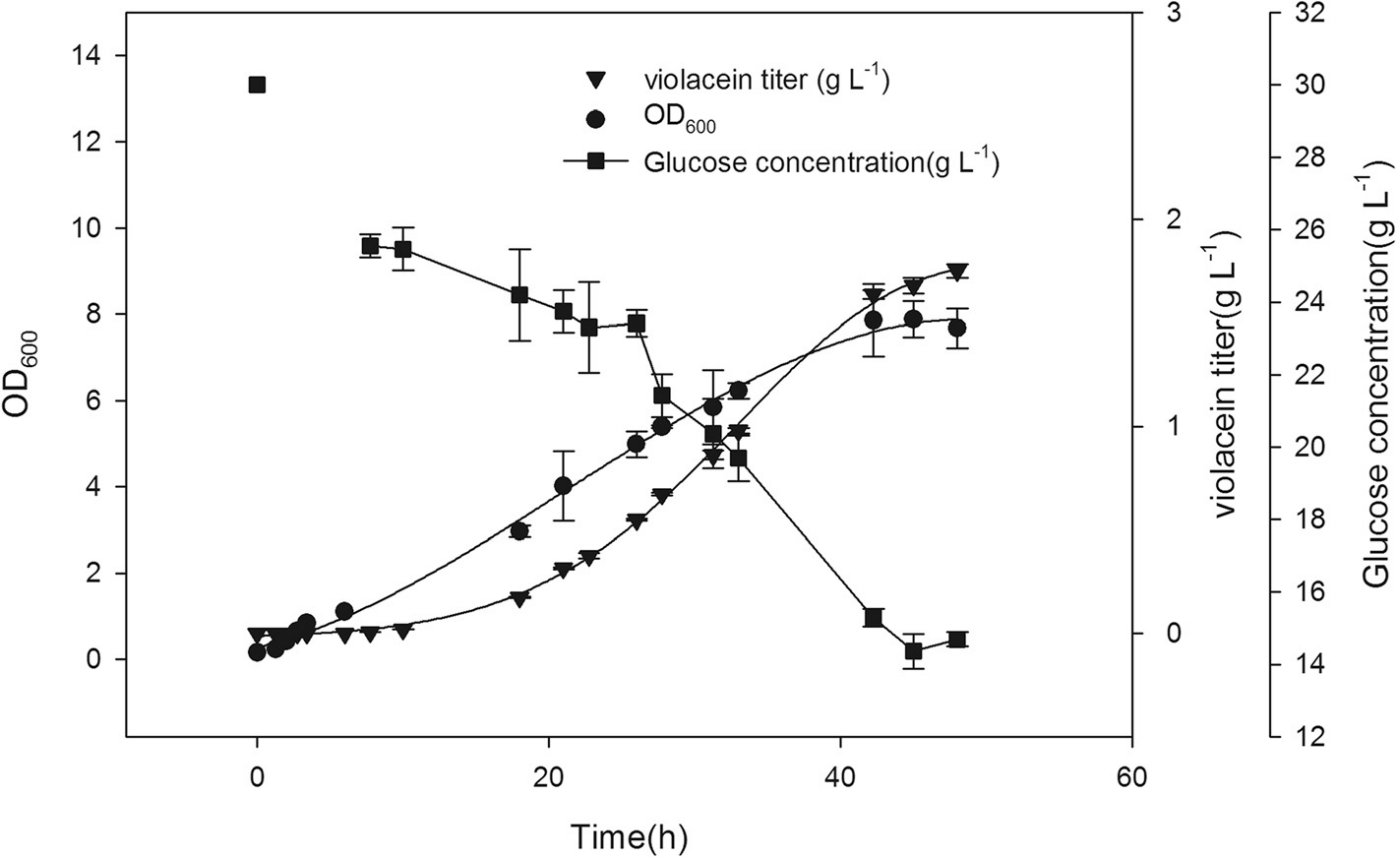
**The time course profiles of batch fermentation of the engineered B2/pED+pVio in 5-L bioreactor containing 2 L of M9-YE medium (30 g L** ^**−1**^ **glucose) under controlled pH and dissolved oxygen level (n=3).**

From the results of the flask culture described earlier, it can be concluded that B2/pED + pVio was the most potential strain for violacein production. This conclusion was further examined by performing fermentation in a 5-L bioreactor. Batch fermentation was conducted by online control of the bioreactor containing 2 L of M9-YE medium with 30 g L^−1^ glucose. When the OD_600_ reached 0.8, the temperature was changed from 37°C to 20°C, and induction was performed with the addition of 0.05 mM IPTG. The time course profiles of OD_600_, glucose concentration, and crude violacein concentration were measured. After induction, violacein started to accumulate during the exponential growth phase, high violacein production stability was noted, and non-violacein-producing variants or low-yield mutants never appeared (data not shown). These findings are in contrast to those reported in a previous study, which demonstrated that recombinant *E. coli* with *vio* gene cluster exhibited high frequency of forming non-violacein-producing variants or low-yield mutants [18]. Furthermore, during the fermentation process, crude violacein was found to be transported extracellularly and occur as crystal-like forms, which might reduce product toxicity and ensure the high titer (Additional file 1: Figures S5 and S6). The secretion of violacein into the broth to form pellets presumably enhanced the high violacein production. As shown in Figure 6, B2/pED + pVio underwent sigmoid growth and the final OD_600_ reached around 7. Violacein began to accumulate after around 10 h of induction, and the final violacein concentration reached 1.75 g L^−1^ at 48 h (with productivity of 36 mg L^−1^ h^−1^ and a yield from glucose = 0.116 g-violacein/g-glucose, 15.1 g L^−1^ glucose consumed), which is 4.48-fold higher than that of *C. freundii* (pCom10vio) under the similar culture conditions without tryptophan addition [13].

## Discussion

Upstream up-regulation to improve precursor supply, a commonly used strategy in metabolic engineering, can result in an increase in the production of target metabolites, especially those that are synthesized by exogenous pathways [20,21]. By using this strategy, we successfully engineered a recombinant *E. coli* for crude violacein production from glucose, with a productivity (36 mg L^−1^ h^−1^) and yield (0.116 g-vioalcein/g-glucose) exceeding most violacein/deoxyviolacein-producing strains under the similar conditions without tryptophan addition reported so far (Table 3). The violacein yield from glucose was estimated to be 0.116 g-violacein/g-glucose, which corresponded to a tryptophan equivalent yield of 0.136 g-tryptophan/g-glucose, as high as 60% of theoretical maximum yield of tryptophan (the theoretical maximum yield of tryptophan toward glucose was 0.227 g-tryptophan/g-glucose) [27]. Although previous studies have shown that violacein production may be inhibited when using glucose as the carbon source [28], in the present study, we achieved a high crude violacein production from glucose. Rodrigues et al. recently reported a successful production of pure violacein and deoxyviolacein by pathway modification from arabinose and glycerol, respectively [15,29]. Since both of violacein and deoxyviolacein have the similar acid/alkali resistance, UV resistance and antibacterial activity [8], we aimed at the pathway engineering for the production of crude violacein (mixture of violacein and deoxyviolacein) directly from glucose in the present study. The pathway engineering demonstrated by Rodrigues et al. [15,29] for production of the pure violacein and deoxyviolacein can be a good strategy for the further work on design of the pure violacein or deoxyviolacein synthesis from different carbon sources.

**Table 3:** **The titers of the reported violacein-producing strains**

**Strain**	**Carbon source**	**Violacein and/or deoxyviolacein titer (g L** ^**−1**^ **)**	**Time (h)**	**Productivity (g L** ^**−1**^ **h** ^**−1**^ **)**	**Bioreactor and fermentation strategy**	**Tryptophan addition**	**Violacein yield**	**Reference**
**Wild strains**								
***C.violaceum***		0.43	36	0.012	Flask culture	0.2 g L^−1^		[22]
		1.1			30-L bioreactor batch fermentation			[3]
***Duganella*** **sp.**		1.62	32	0.050	Flask culture	0.74 g L^−1^		[9]
***J.lividum***	15 g L^−1^ sucrose	0.85	144	0.006	Flask culture	Non	0.057 g g^−1^	[10]
**Recombinant strains**								
***C.freundii*** **/pCOM10vio**		0.75	40	0.019	Flask culture	Non		[6]
		1.68	40	0.042	Flask culture	0.7 g L^−1^		[6]
	5.67 g L^−1^ glycerol	0.47	35	0.013	5-L bioreactor batch fermentation	Non	0.083 g g^−1^	[13]
		0.95	35	0.027	5-L bioreactor batch fermentation	0.7 g L^−1^		[13]
***E.coli*** **BL21(DE3)/pET32avio**		0.12	40	0.003	Flask culture	Non		[6]
***E.coli*** **MG1655-Vio4**		0.71	300	0.002	0.7-L Fed-batch	Non		[14]
***E.coli*** **MG1655-Vio4**	10 g L^−1^ arabinose	0.32	225	0.001	Flask culture	Non		[14]
**MG1655-dVio8**		1.6	200	0.008	Fed-batch	Non	0.032 g g^−1^	[15]
***E.coli*** **Bl21(DE3) B2/pED+pVio**	15.1 g L^−1^ glucose	1.75	48	0.036	5-L bioreactor batch fermentation	Non	0.116 g g^−1^	This research

A multivariate tryptophan supply module was developed to study their influences on downstream violacein production. The strains B8, B6, B2, and B3, which shared the same *trpR* gene knockout, showed the highest tryptophan titers. This was owing to that product of *trpR* gene could mediate the repression of genes relevant to tryptophan biosynthesis included in the *aroG* and *trp* operon [23,30]. As *tnaA* encodes tryptophanase and *pheA* encodes the enzyme for the biosynthesis of tyrosine and phenylalanine, after the knockout of *tnaA* and/or *pheA* tryptophan accumulations exhibited obvious increase but lower than those of strains with *trpR* gene knockout (Figure 2). As these three genes have different influences on the accumulation of tryptophan [24,25,30-32], their combinatorial knockout created multivariate tryptophan titers. After combining with the downstream pathway, B2/pED + pVio was selected as the best producer. The ^13^C labeling analysis demonstrated that the glucose uptake rate was lower for B2/pED + pVio, when compared with that for B1/pVio. We suggested that the PEP, which plays important role in phosphotransferase system for glucose uptake, was consumed as the precursor for tryptophan synthesis (as shown in Additional file 1: Figure S8A), and this resulted in lower glucose uptake rate in B2/pED + pVio. While the carbon flux to tryptophan supply, as a branch to compete the carbon flux to form cell biomass, was increased for B2/pED + pVio than that for B1/pVio on average (Figure 5). Thus lower biomass accumulation in B2/pED + pVio was due to 1) the lower glucose uptake rate and 2) less carbon flux to biomass synthesis. Rodrigues et al. [14] also intensified tryptophan supply for violacein production and used the intracellular tryptophan concentration to identify the supply limitation. In the present study, we used ^13^C labeling analysis with higher resolution [33] to prove the influences of precursor supply on the downstream pathway. To achieve further increase in violacein production, the overexpression of *aroG* [27] and *serA* [14] genes or modification of phosphoenolpyruvate phosphotransferase system for glucose uptake [34] might enable more transfer of the carbon flux from central metabolism to tryptophan synthesis.

We interestingly found that the introduction of downstream violacein biosynthetic pathway obviously enhanced the tryptophan flux. After docking with the downstream pathway, a respective 300- and 15-fold dramatic increase in upstream tryptophan equivalent yield for the two selected strains was observed, quantitatively proving the presence of pulling effect, which means that the carbon flux of upstream was increased due to consumption of metabolites by downstream pathway. In this study, the pulling effect was mainly owing to alleviation of inhibitory regulation, which resulted in decreased loss of carbon flux to the branched pathways and enhanced the tryptophan supply to violacein synthesis. *TnaA, pheA,* and *trpR* play roles in degrading tryptophan, competing carbon flux from tryptophan and inhibiting the transcription of *trp* operon when tryptophan accumulated, while when the downstream pathway for violacein synthesis was docked, less typtophan was accumulated, thus decreasing the effect of knockout of *tnaA, pheA* and *trpR*. This could explain why strain B1 with pED and pVio is already a suitable producer of crude violacein. Previous studies had used multivariate modules with different transcriptional or translational levels to explore a balanced module as precursor supply [19,35]. Thus, the pulling effect on these cases was hard to be found obviously as 1) this effect may not exited in these situations and/or 2) the yields of the precursor and target chemicals were not compared quantitatively to find this effect [19,35]. In the present study, we demonstrated that the downstream violacein biosynthetic pathway could obviously draw the carbon flux of the upstream pathway to produce more precursors from glucose. This kind of interactive pull effect was possible to occur when the accumulated metabolites was consumed effectively by a docked pathway and finally resulted in self-balancing of the long pathway, which dynamically drew more, but not excess, precursor for the downstream pathway to achieve high productivity of the target products.

## Material and methods

### Bacterial strains and culture medium

*E. coli* DH5α (Invitrogen) was used for plasmid construction. The *E. coli* strains and plasmids used in this study are listed in Table 1. Luria-Bertani (LB) medium was used as the complex medium and modified M9-YE medium (17.1 g L^−1^ Na_2_HPO_4_, 3 g L^−1^ KH_2_PO_4_, 0.5 g L^−1^ NaCl, 1 g L^−1^ NH_4_Cl, 1 g L^−1^ Yeast Extract, 1 mM MgSO_4_, and 0.1 mM CaCl_2_) [36] containing glucose as the carbon source was used for tryptophan or violacein production (the glucose concentration was 10 g L^−1^ unless stated). This medium was proved to be sufficient to support growth of all the strains. Kanamycin (50 μg mL^−1^) and/or chloromycetin (34 μg mL^−1^) were added when needed. A 100-mL flask containing 20 mL of M9-YE medium was used for violacein fermentation, and glucose and IPTG (Isopropyl β-D-1-Thiogalactopyranoside) were added as the carbon source and inducer, respectively.

### P1 transduction

JW 3686, JW 4356, and JW 2580 were obtained from the National BioResource Project-*E. coli* (National Institute of Genetics, Mishima, Japan). P1 transduction was used to construct the gene deletion mutants of BL21(DE3) by knocking out *tnaA*, *trpR,* and *pheA* genes separately or in combination to generate eight different gene types of BL21(DE3) [37]. Three makers *ΔtnaA::FRT-kan-FRT*, *ΔtrpR::FRT-kan-FRT* and *ΔpheA::FRT-kan-FRT,* were transduced from *E. coli* K strain JW 3686, JW 4356, and JW 2580, respectively. Then, the helper plasmid pCP20 [38,39] containing a temperature-sensitive origin replication and thermally inducible FLP gene was transformed into this strain, followed by induction to remove the kan maker. Specific primers listed in Additional file 1: Table S1 confirmed the implementation and validation for genetic modification.

### Construction of pACYCDuet*-trpE*^*fbr*^*/trpD* and pRSFDuet*-vio*

The *E. coli* W3110 genomic DNA was extracted with Bacteria Genomic DNA Purification Kit (Tiangen, Beijing) and used as a template for the amplification of *trpED* with Primer Star polymerase (Takara, Japan) and primers NcoI-trpE-F/AflII-trpD-R (Additional file 1: Table S1). Point mutation was conducted (Met293Thr, TCC to TTC; Ser40Leu, ATG to ACG) according to the previous report [40] to reduce feedback inhibition. After digestion by *Nco*I and *Afl*II, the fragment was ligated to *Nco*I/*Afl*II-digested pACYCDuet. The plasmid pCom10-*vio* [6] was used as a template for the amplification of the *vio* gene cluster with two pairs of primers AseI-vio1-F/NotI-vio1-R and Not-vio2-F/XhoI-vio2-R (Additional file 1: Table S1). The two fragments were digested by *Ase*I/*Not*I and *Not*I/*Xho*I, respectively, and ligated to *Nde*I/*Xho*I-digested pRSFDuet. Genes of *trpE*^*fbr*^*/trpD* and *vio* gene cluster were both under the control of IPTG-inducible T7 promoter.

### Analytical method

For the measurement of tryptophan, after fermentation, the supernatants were filtered through 0.22-μm membrane filters for HPLC analysis. The samples were separated in a Inertsil ODS-SP 4.6 × 250 mm column with methanol (A) and 0.05% H_3_PO_4_ in water (B) as the mobile phase and a detector set to a wavelength of 280 nm (0–3 min, 2% A; 3–30 min, 2%–80% A; 30–40 min, 80% A; 40–50 min, 80%–2% A; and 50–60 min, 2% A). For the detection of crude violacein, after fermentation, the culture broth was centrifuged and the crude violacein in the pellet was extracted with ethanol. After total extraction, the pellet was centrifuged again, and the absorbance of the crude violacein solution at 570 nm (Abs_570_) was measured to determine the concentration of crude violacein (Additional file 1: Figure S4) and the ratio between violacein and deoxyviolacein remained constant during the fermentation [29]. Subsequently, the pellet was resuspended in deionized water prior to measurement of the optical density at 600 nm, as described previously [6,13]. The molecular weight of violacein and deoxyviolacein was detected by TOF (Time Of Flight) LC/MS System (Waters, Xevo G2), which demonstrated that the purple pigment was violacein and deoxyviolacein mixture (Additional file 1: Figures S1 and S2). The composition of violacein and deoxyviolacein was determined by HPLC with a Inertsil ODS-SP 4.6 × 150 mm column, 75% methanol in water as the mobile phase, and a detector set to a wavelength of 570 nm (Additional file 1: Figure S3). The crude violacein of this composition was dried to detect the relationship between Abs_570_ and crude violacein concentration (Additional file 1: Figure S4). During fermentation, the residual glucose concentration in the medium was detected by the DNS (dinitrosalicylic acid) method [41], and the pH of the samples was determined by using HORIBA B-712 pH meter.

### The tolerance of *E.coli* to crude violacein

Effect of crude violacein on the growth of *E. coli* was examined by external addition of crude violacein into the culture. The *E. coli* Bl21(DE3) cultures of exponential phase were diluted 1:100 to the fresh M9-YE medium with 10 g L^−1^ glucose. Crude violacein was added at the same time using DMSO (dimethylsulfoxide) as co-solvent to a final concentration of 0 (DMSO only), 0.25, 0.50, 1.00, 2.00 or 4.00 g L^−1^. These cultures were then cultivated at 37°C for 24 hours. Then, the culture broth was centrifuged and the crude violacein in the pellet was extracted with ethanol. After total extraction, the pellet was resuspended in deionized water prior to measurement of the optical density at 600 nm for evaluation of the cell growth.

### ^13^C labeling analysis

Metabolic pathway analysis by ^13^C labeling was performed by using the following protocol based on the method reported by Yang et al. [26]. Metabolites such as D-glucose-6-^13^C (one carbon C6 was ^13^C labeled), D-glucose-^13^C_6_ (six carbons were ^13^C labeled), acyl-CoAs, amino acids, organic acids, and sugar phosphates were obtained from Sigma (St. Louis, MO, USA). The bacterial cells were grown in M9-YE medium with ^12^C glucose (5 g L^−1^) as the carbon source. In the middle of the exponential phase (OD_600_ = 0.70–0.80), 8 mL of the culture were extracted and rapidly passed through the membrane filter (0.22 μm, 47 mm, Sartorius) using a pipette. The filter was immediately removed and placed on an agar plate of M9-YE medium with ^12^C glucose for 20 min, and then transferred to another agar plate with the same concentration of ^13^C glucose. At defined time points, the filter was immediately transferred to a 15-mL tube with liquid nitrogen for quenching. The sample was stored at −80°C for subsequent extraction.

The metabolites extraction method was modified according to the previous procedure reported by Yang et al. [26]. Briefly, 2 mL of hot extraction buffer (60%, v/v ethanol/water, 1 mM EDTANa_2_, and 5 mM ammonium acetate) were added to the sample and incubated at 80°C for 3 min. The extracted suspension was centrifuged at 5000 × *g* for 5 min to remove cell debris. Subsequently, the suspension was centrifuged again at 13,000 × *g* for 15 min, and the supernatant was dried in a vacuum centrifuge (Christ, RVC 2–18 CD plus, Germany). For LC-MS analysis, the dried sample was dissolved in 100 μL of acetonitrile/water (1:1, v/v) and analyzed on an Agilent 6460 system. For separation on an Acquity UPLC BEH Amide (Waters) column, the mobile phase A was acetonitrile/water (2:98,v/v), 0.2% formic acid, and 0.1% ammonium hydroxide, and the mobile phase B was acetonitrile/water (95:5,v/v), 0.1% formic acid, and 0.075% ammonium hydroxide. For the metabolites detected in the positive ESI mode, the following linear gradient was used: 0–0.5 min, 100% B; 0.5–5.5 min, 100%–81% B; 5.5–11 min, 81%–72% B; 11–16 min, 72%–40% B; 16–17 min, 40%–100% B; and 17–29 min, 100% B. For the metabolites detected in the negative mode, the following linear gradient was used: 0–4 min, 100% B; 4–21 min, 100%–72% B; 21–26 min, 72%–40% B; 26–27 min, 40%–100% B; and 27–40 min, 100% B. The total run time was 29 and 40 min at 0.2 mL min^−1^, respectively. The injection volume was 10 μL for both methods. The LC-MS/MS experiments were conducted as described previously [26]. The dwell time for each MRM (multiple reaction monitoring) transition was 16 s. All the peaks were integrated using the Qualitative Analysis B.05.00 software. The total ^13^C incorporation of a metabolite with *N* carbon atoms was calculated by normalizing to its total carbon number according to the previous protocol [26] as shown in Eq. (1) and Eq. (2).

1 $$ {M}_i\left(\%\right)=\frac{m_i}{\sum_{j=0}^n{m}_j} $$

2 $$ \mathrm{Tota}{\mathrm{l}}^{13}\mathrm{C}\;\mathrm{incorporation}\;\left(\%\right)=\frac{\sum_{i=}^N\left(i\kern0.5em \times \kern0.5em {M}_i\right)}{N} $$

where *m*_*i*_ represented the isotopic abundance for a metabolite in which the *i*^13^C atoms were incorporated and *n* represented the maximum number of ^13^C atoms incorporated.

### C-mol biomass, tryptophan, and tryptophan equivalent yields from glucose

The medium used in this experiment was M9-YE with 10 g L^−1^ glucose and 0.05 mM IPTG was used for induction. Glucose consumption was calculated by detecting the concentration of glucose in the medium (the carbon source from yeast extraction was ignored). The dry cell weight was calculated using Eq. (3) according to OD_600_. The ash content and composition of the biomass was estimated to 5.5% and CH_1.8_O_0.5_ N_0.2_ (molecular weight of biomass (*M*_*biomass*_) = 24.6) as *E. coli* K-12, respectively [33]. For the tryptophan producer, the actual tryptophan concentration was mainly contributed by the extracellular concentration as detected by HPLC, and hence, intracellular tryptophan concentration could be ignored. The peak areas of violacein and deoxyviolacein produced in the present study were similar (Additional file 1: Figure S3), and their extinction coefficients were 0.074 and 0.048 L mg^−1^ cm^−1^ respectively [29]. As no pure violacein and deoxyviolacein were available, violacein and deoxyviolacein were estimated to account for 39% and 61% of the crude violacein, respectively. The tryptophan concentration equivalent for the violacein producers was calculated according to violacein titer as expressed by Eq. (4). Incidentally, in all of the violacein producers, the residual tryptophan could not be detected (data not shown), which indicated that the tryptophan synthesized by the upstream pathway was quickly converted to violacein inside the cells. Thus, the C-mol biomass, tryptophan, and tryptophan equivalent yields were calculated according to Eq. (5), (6), and (7), respectively.

3 $$ \mathrm{Dry}\;\mathrm{cell}\kern0.5em \mathrm{weight}\kern0.5em \left(\mathrm{g}\kern0.5em {\mathrm{L}}^{\hbox{-} 1}\right)=0.331\kern0.5em \times \kern0.5em 0{\mathrm{D}}_{600}\hbox{-} 0.0057 $$

4 $$ \begin{array}{l}\mathrm{Tryptophan}\;\mathrm{concentration}\;\mathrm{equivalent}\;\left(\mathrm{g}\;{\mathrm{L}}^{\hbox{-} 1}\right)\\ {}=\left(\frac{\mathrm{crude}\kern0.5em \mathrm{violacein}\kern0.5em \mathrm{concentration}\times 39\%}{M_{violacein}}\kern0.5em +\frac{\mathrm{crude}\kern0.5em \mathrm{violacein}\kern0.5em \mathrm{concentration} \times 61\%}{M_{deoxviolacein}}\right)\times {M}_{tryptophan}\times \kern0.5em 2\end{array} $$

5 $$ \mathrm{C}\hbox{-}\;\mathrm{mol}\;\mathrm{biomass}\;\mathrm{yield}=\frac{\mathrm{Dry}\kern0.5em \mathrm{cell}\kern0.5em \mathrm{weight}\kern0.5em \left(\mathrm{g}\;{\mathrm{L}}^{\hbox{-} 1}\right)\kern0.5em \times \kern0.5em \left(1\hbox{-} 5.5\%\right)/\kern0.1em {M}_{biomass}}{\mathrm{Glucose}\kern0.5em \mathrm{consumption}\kern0.5em \left(\mathrm{g}\;{\mathrm{L}}^{\hbox{-} 1}\right)/{M}_{glu \cos e}\times \kern0.5em 6} $$

6 $$ \mathrm{C}\hbox{-}\;\mathrm{mol}\;\mathrm{tryptophan}\;\mathrm{yield}=\frac{\mathrm{tryptophan}\ \mathrm{concentration}\kern0.5em \left(\mathrm{g}\;{\mathrm{L}}^{\hbox{-} 1}\right)\kern0.5em \times /\kern0.1em {M}_{tryptophan}\kern0.5em \times \kern0.5em 11}{\ \mathrm{Glucose}\kern0.5em \mathrm{consumption}\kern0.5em \left(\mathrm{g}\;{\mathrm{L}}^{\hbox{-} 1}\right)/{M}_{glu \cos e}\times \kern0.5em 6} $$

7 $$ \mathrm{C}\hbox{-}\;\mathrm{mol}\;\mathrm{tryptophan}\;\mathrm{equivalent}\;\mathrm{yield}=\frac{\mathrm{tryptophan}\ \mathrm{concentration}\ \mathrm{equivalent}\kern0.5em \left(\mathrm{g}\;{\mathrm{L}}^{\hbox{-} 1}\right)/\kern0.1em {M}_{tryptophan}\kern0.5em \times \kern0.5em 11}{\mathrm{Glucose}\kern0.5em \mathrm{consumption}\kern0.5em \left(\mathrm{g}\;{\mathrm{L}}^{\hbox{-} 1}\right)/{M}_{glu \cos e}\times \kern0.5em 6} $$

where *M*_*violacein*_, *M*_*deoxyviolacein*_, *M*_*tryptophan*_, *M*_*biomass*_ and *M*_*glucose*_ represent the molecular weight of violacein, deoxyviolacein, tryptophan, biomass and glucose, respectively.

### Fermentation kinetics of the engineered strain B2*/*pED+pVio

The engineered strain B2/pED+pVio was incubated at 37°C and 200 rpm overnight in LB broth with kanamycin (50 μg mL^−1^) and chloromycetin (34 μg mL^−1^). Subsequently, 5% inoculum (100 mL for 2 L) was added into 2 L of fresh M9-YE medium containing 30 g L^−1^ glucose in a 5-L stirred tank bioreactor (Sartorius, Germany). The temperature was initially controlled at 37±0.1°C and then at 20±0.1°C after the induction according to our previous report [6,8-10,13]. The induction was performed with 0.05 mM IPTG when the OD_600_ value of the culture reached 0.8 and the temperature of the culture was shifted to 20°C. The dissolved oxygen level was controlled above 25±5% with Inpro 6800/12/320 O_2_ sensor (Mettler Toledo). The pH of the culture was controlled at 7.0±0.5 using 405-DPAS-SC-K8S/325 pH electrode and 10 M NaOH/3 M HCl. The samples were withdrawn during fermentation to measure the concentration of glucose, crude violacein, and cell density (OD_600_).

## Additional file

Additional file 1: Table S1. Primers and their sequence. **Table S2.** Tryptophan titers and pH values of B8/pED in different medium. **Table S3.** Violacein titer and pH of B8/pED+pVio in different medium. **Table S4.** Optimization of glucose concentration and IPTG induction concentration for violacein production. **Table S5.** Comparison of B1/pVio and B1/pACYCDuet +pVio for violacein fermentation. **Figure S1.** Molecular mass of violacein. **Figure S2.** Molecular mass of deoxyviolacein. **Figure S3.** HPLC of crude violacein (violacein and deoxyviolacein mixture). **Figure S4.** The relationship between Absorbance at 570nm and concentration of crude violacein. **Figure S5** Microscopic photos of *E. coli* (left) and solid particle of violacein (left, the purple one which was circled in dashed line) during fermentation **Figure S6.** Separation of medium and bacterial cells of fermentation broth (left: before centrifugation; right: after centrifugation). **Figure S7A.** The ^13^C incorporation rate of different metabolites in B2/pED+pVio (Red) and B1/pVio (Blue) according to different time periods. **Figure S7B.** Pathway metabolic flux change of B2/pED+pVio compared with B1/pVio generated generated from Figure S7A. **Figure S8.** Theoretical maximum yield of tryptophan toward glucose in absence of constant maintenance energy requirement. The numbers indicate the relative flux carried by the reactions. **8A**, the theoretical maximum yield of tryptophan generated under common situation (0.20 molar yield); **8B**, the theoretical maximum yield of tryptophan generated when the PTS is not taken into account (0.40 molar yield). **8C**, the theoretical maximum yield of tryptophan generated when pyruvate is recycled back to PEP (0.59 molar yield).

## Abbreviations

E4P: Erythrose 4-phosphate
PEP: Phosphoenolpyruvate
DS: 3-deoxy-D-arabinoheptulosonate 7-phosphate synthase
DAHP: 3-deoxy-D-arabinoheptulosonate 7-phosphate
CM: Chorismate mutase
CA: Chorismic acid
Phe: Phenylalanine
Tyr: Tyrosine
Trp: Tryptophan
Vio: Violacein
Devio: Deoxyviolacein
G-6-P/F-6-P: Fructose-6-phosphate/glucose-6-phosphate
F-1,6-bis-P: Fructose-1,6-bisphosphate
2-PG/3-PG: 2-phosphoglycerate/3-phosphoglycerate

## References

[1] Ryan KS, Drennan CL (2009). Divergent pathways in the biosynthesis of bisindole natural products. Chem Biol.

[2] Nelson Durán CFMM (2001). Chromobacterium violaceum: a review of pharmacological and industiral perspectives. Crit Rev Microbiol.

[3] Durán N, Justo GZ, Ferreira CV, Melo PS, Cordi L, Martins D (2007). Violacein: properties and biological activities. Biotechnol Appl Biochem.

[4] Rettori D, Durán N (1998). Production, extraction and purificationof violacein: an antibiotic pigment producedby Chromobacterium violaceum. World J Microb Biot.

[5] Durán N, Antonio RV, Haun M, Pilli RA (1994). Biosynthesis of a trypanocide by Chromobacterium violaceum. World J Microb Biot.

[6] Jiang PX, Wang HS, Zhang C, Lou K, Xing XH (2010). Reconstruction of the violacein biosynthetic pathway from Duganella sp B2 in different heterologous hosts. Appl Microbiol Biotechnol.

[7] Bromberg N, Durán N (2001). Violacein transformation by peroxidases and oxidases: implications on its biological properties. J Mol Catal B-Enzym.

[8] Jiang PX, Wang HS, Xiao S, Fang MY, Zhang RP, He SY, Lou K, Xing XH (2012). Pathway redesign for deoxyviolacein biosynthesis in Citrobacter freundii and characterization of this pigment. Appl Microbiol Biotechnol.

[9] Wang H, Jiang P, Lu Y, Ruan Z, Jiang R, Xing X-H, Lou K, Wei D (2009). Optimization of culture conditions for violacein production by a new strain of Duganella sp. B2. Biochem Eng J.

[10] Lu Y, Wang L, Xue Y, Zhang C, Xing X-H, Lou K, Zhang Z, Li Y, Zhang G, Bi J, Su Z (2009). Production of violet pigment by a newly isolated psychrotrophic bacterium from a glacier in Xinjiang, China. Biochem Eng J.

[11] Patijanasoontorn B, Boonma P, Wilailackana C, Sitthikesorn J, Lumbiganon P, Chetchotisakd P, Noppawinyoowong C, Simajareuk K (1992). Hospital acquired Janthinobacterium lividum septicemia in Srinagarind Hospital. J Med Assoc Thai.

[12] Ti TY, Tan WC, Chong AP, Lee EH (1993). Nonfatal and fatal infections caused by Chromobacterium violaceum. Clin Infect Dis.

[13] Yang C, Jiang P, Xiao S, Zhang C, Lou K, Xing X-H (2011). Fed-batch fermentation of recombinant Citrobacter freundii with expression of a violacein-synthesizing gene cluster for efficient violacein production from glycerol. Biochem Eng J.

[14] Rodrigues AL, Trachtmann N, Becker J, Lohanatha AF, Blotenberg J, Bolten CJ, Korneli C, de Souza Lima AO, Porto LM, Sprenger GA, Wittmann C (2013). Systems metabolic engineering of Escherichia coli for production of the antitumor drugs violacein and deoxyviolacein. Metab Eng.

[15] Rodrigues AL, Becker J, de Souza Lima AO, Porto LM, Wittmann C (2014). Systems metabolic engineering of Escherichia coli for gram scale production of the antitumor drug deoxyviolacein from glycerol. Biotechnol Bioeng.

[16] Pemberton J, Vincent K, Penfold R (1991). Cloning and heterologous expression of the violacein biosynthesis gene cluster from Chromobacterium violaceum. Curr Microbiol.

[17] Balibar CJ, Walsh CT (2006). In vitro biosynthesis of violacein from l-Tryptophan by the enzymes VioA−E from Chromobacterium violaceum†. Biochemistry.

[18] Sanchez C, Brana AF, Mendez C, Salas JA (2006). Reevaluation of the violacein biosynthetic pathway and its relationship to indolocarbazole biosynthesis. Chembiochem.

[19] Ajikumar PK, Xiao WH, Tyo KE, Wang Y, Simeon F, Leonard E, Mucha O, Phon TH, Pfeifer B, Stephanopoulos G (2010). Isoprenoid pathway optimization for Taxol precursor overproduction in Escherichia coli. Science.

[20] Chou HH, Keasling JD (2013). Programming adaptive control to evolve increased metabolite production. Nat Commun.

[21] Yoon SH, Lee YM, Kim JE, Lee SH, Lee JH, Kim JY, Jung KH, Shin YC, Keasling JD, Kim SW (2006). Enhanced lycopene production in Escherichia coli engineered to synthesize isopentenyl diphosphate and dimethylallyl diphosphate from mevalonate. Biotechnol Bioeng.

[22] Mendes AS, De Carvalho JE, Duarte MCT, Duran N, Bruns RE (2001). Factorial design and response surface optimization of crude violacein for Chromobacterium violaceum production. Biotechnol Lett.

[23] Ikeda M (2006). Towards bacterial strains overproducing L-tryptophan and other aromatics by metabolic engineering. Appl Microbiol Biotechnol.

[24] Zhao ZJ, Zou C, Zhu YX, Dai J, Chen S, Wu D, Wu J, Chen J (2011). Development of L-tryptophan production strains by defined genetic modification in Escherichia coli. J Ind Microbiol Biotechnol.

[25] Gu P, Yang F, Kang J, Wang Q, Qi Q (2012). One-step of tryptophan attenuator inactivation and promoter swapping to improve the production of L-tryptophan in Escherichia coli. Microb Cell Fact.

[26] Yang S, Matsen JB, Konopka M, Green-Saxena A, Clubb J, Sadilek M, Orphan VJ, Beck D, Kalyuzhnaya MG (2013). Global molecular analyses of methane metabolism in methanotrophic alphaproteobacterium, methylosinus trichosporium OB3b. Part II. Metabolomics and 13C-Labeling study. Front Microbiol.

[27] Berry A (1996). Improving production of aromatic compounds in Escherichia coli by metabolic engineering. Trends Biotechnol.

[28] Pantanella F, Berlutti F, Passariello C, Sarli S, Morea C, Schippa S (2007). Violacein and biofilm production in Janthinobacterium lividum. J Appl Microbiol.

[29] Rodrigues AL, Gocke Y, Bolten C, Brock NL, Dickschat JS, Wittmann C (2012). Microbial production of the drugs violacein and deoxyviolacein: analytical development and strain comparison. Biotechnol Lett.

[30] Tribe DE, Pittard J (1979). Hyperproduction of tryptophan by Escherichia coli: genetic manipulation of the pathways leading to tryptophan formation. Appl Environ Microbiol.

[31] Aiba S, Tsunekawa H, Imanaka T (1982). New approach to tryptophan production by Escherichia coli: genetic manipulation of composite plasmids in vitro. Appl Environ Microbiol.

[32] Chan E-C, Tsai H-L, Chen S-L, Mou D-G (1993). Amplification of the tryptophan operon gene in Escherichia coli chromosome to increase l-tryptophan biosynthesis. Appl Microbiol Biotechnol.

[33] Stephanopoulos GN, Aristidou AA, Nielsen J (1998). Metabolic engineering–principles and methodologies academic press.

[34] Patnaik R, Liao JC (1994). Engineering of Escherichia coli central metabolism for aromatic metabolite production with near theoretical yield. Appl Environ Microbiol.

[35] Comba S, Arabolaza A, Gramajo H (2012). Emerging engineering principles for yield improvement in microbial cell design. Comput Struct Biotechnol J.

[36] Chen Y, Xing X-H, Ye F, Kuang Y, Luo M (2007). Production of MBP–HepA fusion protein in recombinant Escherichia coli by optimization of culture medium. Biochem Eng J.

[37] Baba T, Ara T, Hasegawa M, Takai Y, Okumura Y, Baba M, Datsenko KA, Tomita M, Wanner BL, Mori H (2006). Construction of Escherichia coli K-12 in-frame, single-gene knockout mutants: the Keio collection. Mol Syst Biol.

[38] Datsenko KA, Wanner BL (2000). One-step inactivation of chromosomal genes in Escherichia coli K-12 using PCR products. Proc Natl Acad Sci U S A.

[39] Cherepanov PP, Wackernagel W (1995). Gene disruption in Escherichia coli: TcR and KmR cassettes with the option of Flp-catalyzed excision of the antibiotic-resistance determinant. Gene.

[40] Spraggon G, Kim C, Nguyen-Huu X, Yee M-C, Yanofsky C, Mills SE (2001). The structures of anthranilate synthase of Serratia marcescens crystallized in the presence of (i) its substrates, chorismate and glutamine, and a product, glutamate, and (ii) its end-product inhibitor, l-tryptophan. Proc Natl Acad Sci USA.

[41] Miller GL (1959). Use of dinitrosalicylic acid reagent for determination of reducing sugar. Anal Chem.

