# Supplementary material for: High crude violacein production from glucose by Escherichia coli engineered with interactive control of tryptophan pathway and violacein biosynthetic pathway
Source: Microb Cell Fact. 2015 Jan 16;14:8. doi: 10.1186/s12934-015-0192-x (PMC4306242; doi:10.1186/s12934-015-0192-x)
Supplement: Additional file 1: Table S1. — Primers and their sequence. Table S2. Tryptophan titers and pH values of B8/pED in different medium. Table S3. Violacein titer and pH of B8/pED+pVio in different medium. Table S4. Optimization of glucose concentration and IPTG induction concentration for violacein production. Table S5. Comparison of B1/pVio and B1/pACYCDuet +pVio for violacein fermentation. Figure S1. Molecular mass of violacein. Figure S2. Molecular mass of deoxyviolacein. Figure S3. HPLC of crude violacein (violacein and deoxyviolacein mixture). Figure S4. The relationship between Absorbance at 570nm and concentration of crude violacein. Figure S5 Microscopic photos of E. coli (left) and solid particle of violacein (left, the purple one which was circled in dashed line) during fermentation Figure S6. Separation of medium and bacterial cells of fermentation broth (left: before centrifugation; right: after centrifugation). Figure S7A. The 13C incorporation rate of different metabolites in B2/pED+pVio (Red) and B1/pVio (Blue) according to different time periods. Figure S7B. Pathway metabolic flux change of B2/pED+pVio compared with B1/pVio generated generated from Figure S7A. Figure S8. Theoretical maximum yield of tryptophan toward glucose in absence of constant maintenance energy requirement. The numbers indicate the relative flux carried by the reactions. 8A, the theoretical maximum yield of tryptophan generated under common situation (0.20 molar yield); 8B, the theoretical maximum yield of tryptophan generated when the PTS is not taken into account (0.40 molar yield). 8C, the theoretical maximum yield of tryptophan generated when pyruvate is recycled back to PEP (0.59 molar yield). [file 12934_2015_192_MOESM1_ESM.docx]

**Supporting Material**

**Primers used in this research**

Table S1 primers and their sequence.

| **primers** |  |
| --- | --- |
| **Primers for genes knockout** |  |
| tnaA-F | 5'-CGG AAT ATT CCC CAT GGT GCA ATA G-3' |
| tnaA-R | 5'-CTG GCG AAT TAA TCG GTA TAG CAG ATG TA-3' |
| trpR-F | 5'-GCT TTC AAC AAC CAA TGG TGG GAT CTT AG-3' |
| trpR-R | 5'-CGG TAT TGA TGA GAT TGG TCG TAA GGA A-3' |
| pheA-F | 5'-AAA CAG AAT GCG AAG ACG AAC AAT AAG GC-3' |
| pheA-R | 5'-TTG CGT CGG GTG ATG CGT GAA TCT TAC-3' |
| **Primers for plasmids construction** |  |
| NcoI-trpE-F | 5'-CTG TCC ATG GGC ATG CAA ACA CAA AAA CCG ACT CTC GAAC-3' |
| AflII-trpD-R | 5'-ATC TTA AGT TAC CCT CGT GCC GCC AGT GC-3' |
| AseI-vio1-F | 5'-GGATC ATTAA TGACA AATTA TTCTG ACATT TGCAT AG-3' |
| NotI-vio1-R | 5'-AAGAG TGGAC TTGGC GGCCG CTTCG ACCTG-3' |
| Not-vio2-F | 5'-TACAT GACTC AGGTC GAAGC GGCCG CCAAG-3' |
| XhoI-vio2-R | 5'-GGAAT GTCCT CGAGT TCCGA CACGA AAACG CTGGC-3' |

**Selection of culture medium for tryptophan and violacein production.**

B8/pED was cultured in five kinds of different culture medium for tryptophan production. The engineered strain was incubated at 37°C at 200 rpm overnight in LB broth with chloromycetin (50μg mL^-1^) before inoculation of 5% to fresh media containing 10 g/L glucose( except for TP medium) in the 48-well deep microplates. After incubation for 2 hours at 37°C, all the cultures were induced with 0.1mM IPTG followed by cultured at 20°C for 48h. After fermentation, the OD600 was first measured with 96-well microplate and microplate reader (Tecan, Switzerland). Then the culture was centrifuged, and the supernatant was filtered for HPLC and pH measurement. From the result in Table S2, M9-YE was the best medium among the five selected medium with highest tryptophan production. MOPs minimal, MOPs Minimal+NH4Cl and M9-YE were further evaluated for violacein production.

Table S2 Tryptophan titers and pH values of B8/pED in different medium.

| Medium | Tryptophan titer(mg/L) | final OD600 | initial pH | final pH |
| --- | --- | --- | --- | --- |
| TP medium | 40.3±0.001 | 1.88±0.01 | 7.3 | 7.2 |
| MOPs Minimal | 84.3±0.002 | 1.66±0.11 | 7.1 | 6.6 |
| MOPs Minimal+NH4Cl | 88.5±0.001 | 1.71±0.15 | 7.1 | 6.6 |
| MOPs Minimal+NH4Cl+Na2HPO4 | 20.1±0.011 | 1.58±0.11 | 7.2 | 6.8 |
| M9-YE | 107.3±0.006 | 2.09±0.03 | 7 | 5.9 |

B8/pED+pVio was used for evaluation of violacein-production medium. Inoculum and induction were the same as described for tryptophan production except that the antibiotics were kanamycin (50μg mL^-1^) and chloromycetin (34μg mL^-1^). After induction, the culture was cultured at 20°C for 48 hours. After fermentation, the precipitant was first extracted by ethanol, and then the supernatant was used for Abs570 measurement. And the precipitant was suspended with deionized water followed by OD600 measurement. From table S3, engineered strain B8/pED+pVio cultured in M9-YE medium had the highest violacein production.

Table S3 Violacein titer and pH of B8/pED+pVio in different medium.

| Medium | violacein titer(mg/L) | final OD600 | initial pH | final pH |
| --- | --- | --- | --- | --- |
| MOPs Minimal | 41.6±2.7 | 0.35±0.01 | 7.1 | 7.1 |
| MOPs Minimal+NH4Cl | 39.0±3.1 | 0.36±0.01 | 7.1 | 7 |
| M9-YE | 82.8±5.1 | 0.44±0.02 | 7 | 7 |

Then the influence of glucose concentration on violacein production was evaluated with B1/pED+pVio. From the result in Table S4, M9-YE with variant glucose concentration will not significantly influence the violacein production and final OD value (Table S3) of B1/pED+pVio. As for different culture system, enough glucose concentration was supplied for the growth (10g/L for flask culture, and 30g/L for fermentation in bioreactor). The glucose concentration was 5 g/L in ^13^C labeling experiments due to the short time culture and high price of ^13^C labeled glucose (Figure 5). On the other hand, the IPTG concentration had influence on violacein titers (Table S4), so 0.05mM IPTG was used for the induction when tryptophan or violacein fermentation carried out in flask culture or bioreactor fermentation.

Table S4 Optimization of glucose concentration and IPTG induction concentration for violacein production.

| Glucose concentration  (g/L) | Violacein titer (g/L) | final OD600 |
| --- | --- | --- |
| 5 | 0.299±0.013 | 1.35±0.04 |
| 10 | 0.277±0.009 | 1.37±0.03 |
| 20 | 0.304±0.003 | 1.39±0.06 |
| 30 | 0.327±0.003 | 1.41±0.05 |
| IPTG  (mM) | **Violacein titer (g/L)** | **final OD600** |
| 0.05 | 0.532±0.009 | 1.61±0.34 |
| 0.10 | 0.386±0.020 | 0.95±0.14 |
| 0.20 | 0.271±0.017 | 1.14±0.11 |
| 0.30 | 0.241±0.014 | 1.37±0.07 |
| 0.40 | 0.248±0.015 | 1.48±0.07 |

**Influence of empty plasmid on violacein titers and final OD600.**

Table S5 Comparison of B1/pVio and B1/pACYCDuet +pVio for violacein fermentation.

| Strain | Violacein titer (g/L) | final OD600 |
| --- | --- | --- |
| B1/pVio | 0.153±0.005 | 5.96±0.06 |
| B1/pACYCDuet+pVio | 0.160±0.013 | 5.42±0.35 |

**Identity and quantification of crude violacein.**


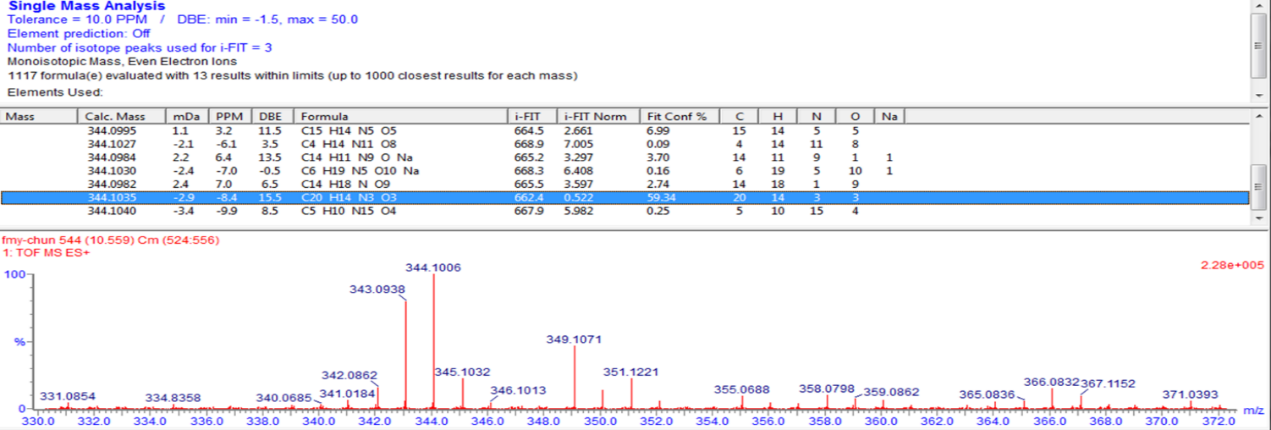


Figure S1 Molecular mass of violacein.


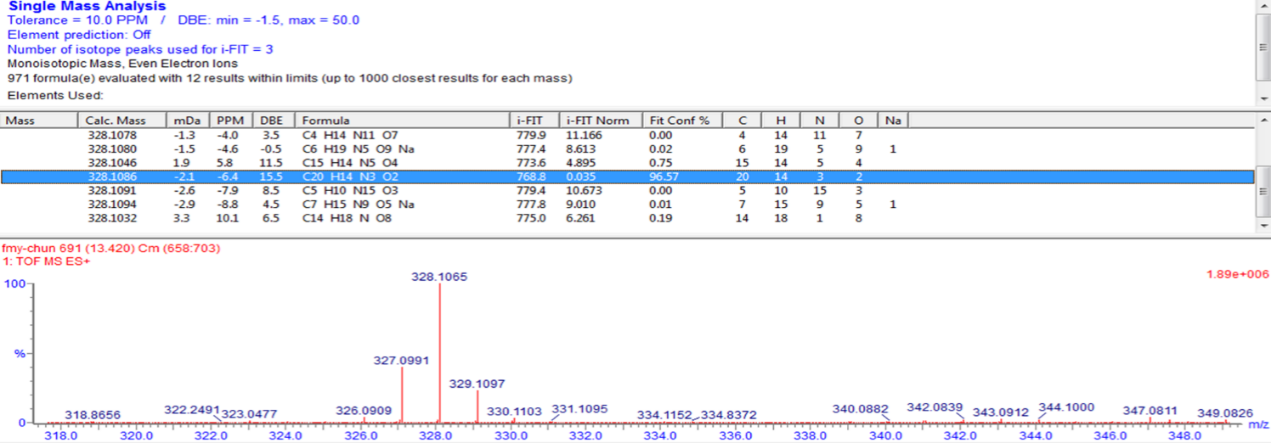


Figure S2 Molecular mass of deoxyviolacein.

Figure S3 HPLC of crude violacein (violacein and deoxyviolacein mixture). The first peak was violacein at 4.7 min, and the second peak was deoxyviolacein at 7.9 min.

Figure S4 The relationship between Absorbance at 570nm and concentration of crude violacein.

**Excretion of crude violacein during fementation.**

Figure S5 showed the microscopic picture of bacteria and crude violacein particles during fermentation. The method used in Figure S6 was modified from Klingenberg. M et.al[1], 800ul of 20% HClO4 was used as lowest layer, 2ml of silicone oil was used as middle layer, 3ml of samples was added slowly as first layer, and then the tube was centrifuged at 6000g for 5min. After centrifugation, the cells went through silicone oil and the medium stayed at the first layer. The tube at right was separation of bacteria and medium after centrifugation, it was found that most violacein stayed in the medium and was separated from bacteria, demonstrating that violacein was extracellular.


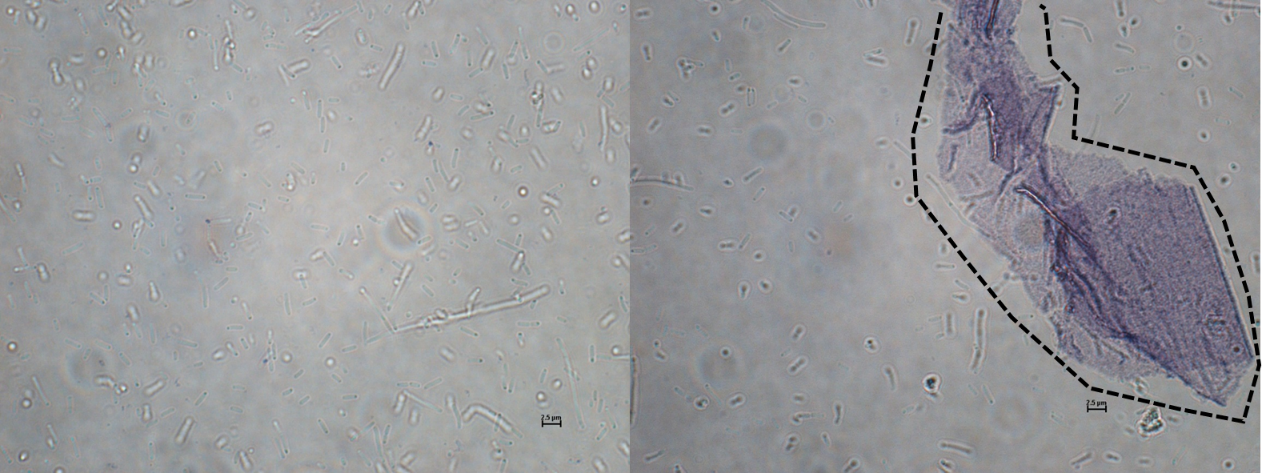


Figure S5 Microscopic photos of *E. coli* (left) and solid particle of violacein(left, the purple one which was circled in dashed line) during fermentation. Bar indicated 2.5 μm.


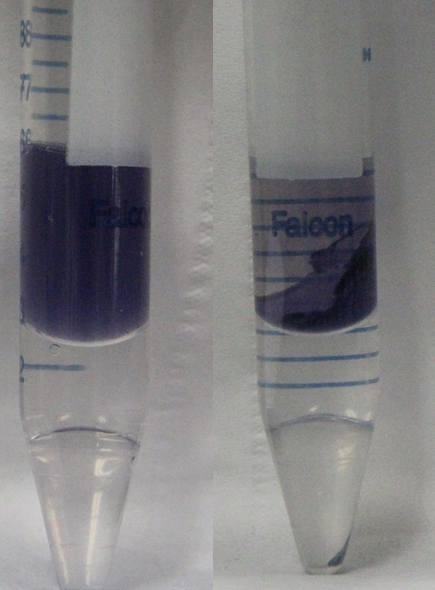


Figure S6 Separation of medium and bacterial cells of fermentation broth (left: before centrifugation ; right: after centrifugation).

**Time course of ^13^C labeling for different intermediates and the change of pathway metabolic flux.**


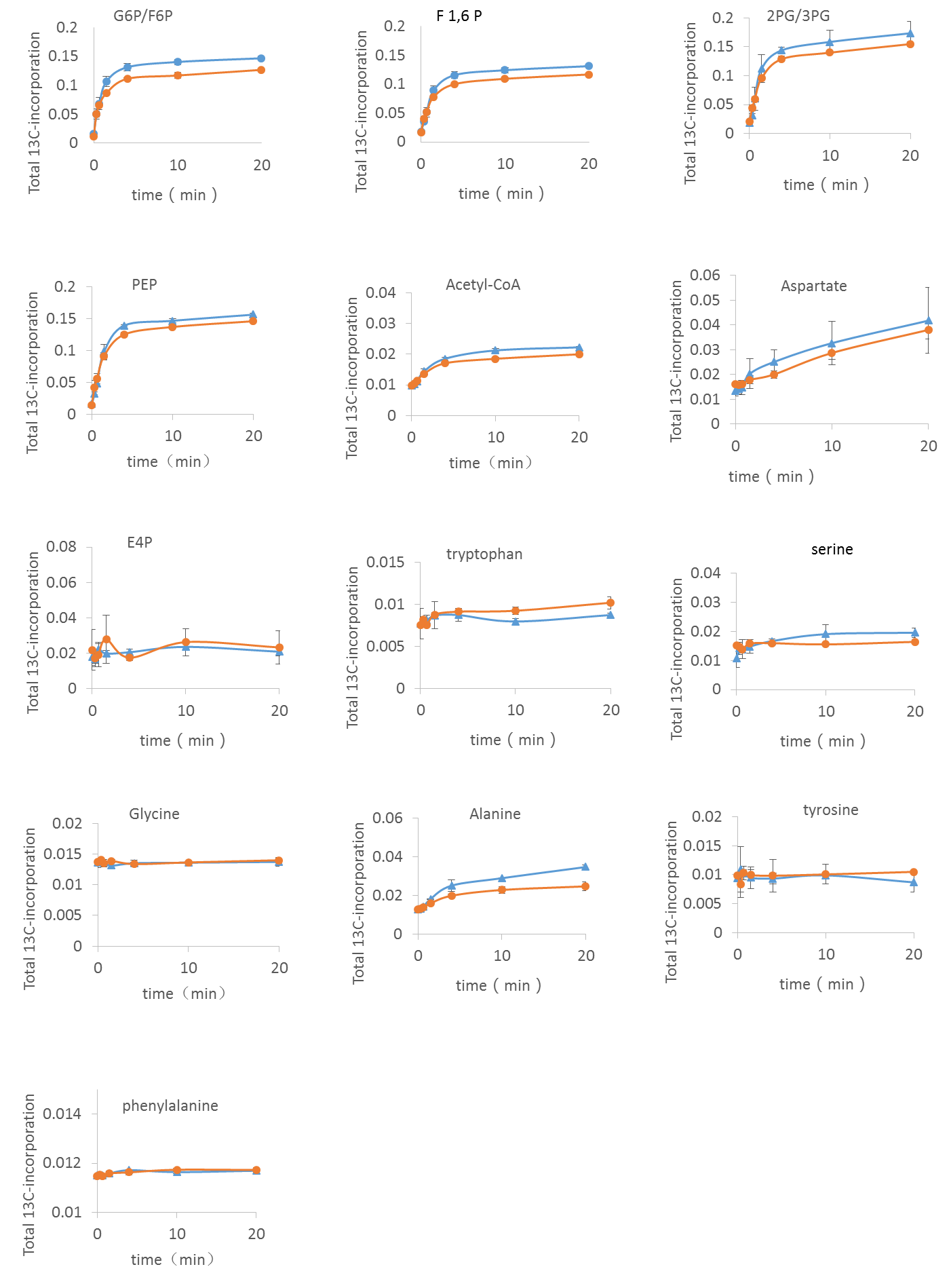


Figure S7A The ^13^C incorporation rate of different metabolites in B2/pED+pVio (Red) and B1/pVio (Blue) according to different time periods. G6P/F6P, fructose-6-phosphate/glucose-6-phosphate; F1,6P, Fructose-1,6-bisphosphate; 2PG/3PG, 2-phosphoglycerate/3-phosphoglycerate; PEP, Phosphoenolpyruvate; E4P, Erythrose 4-phosphate.


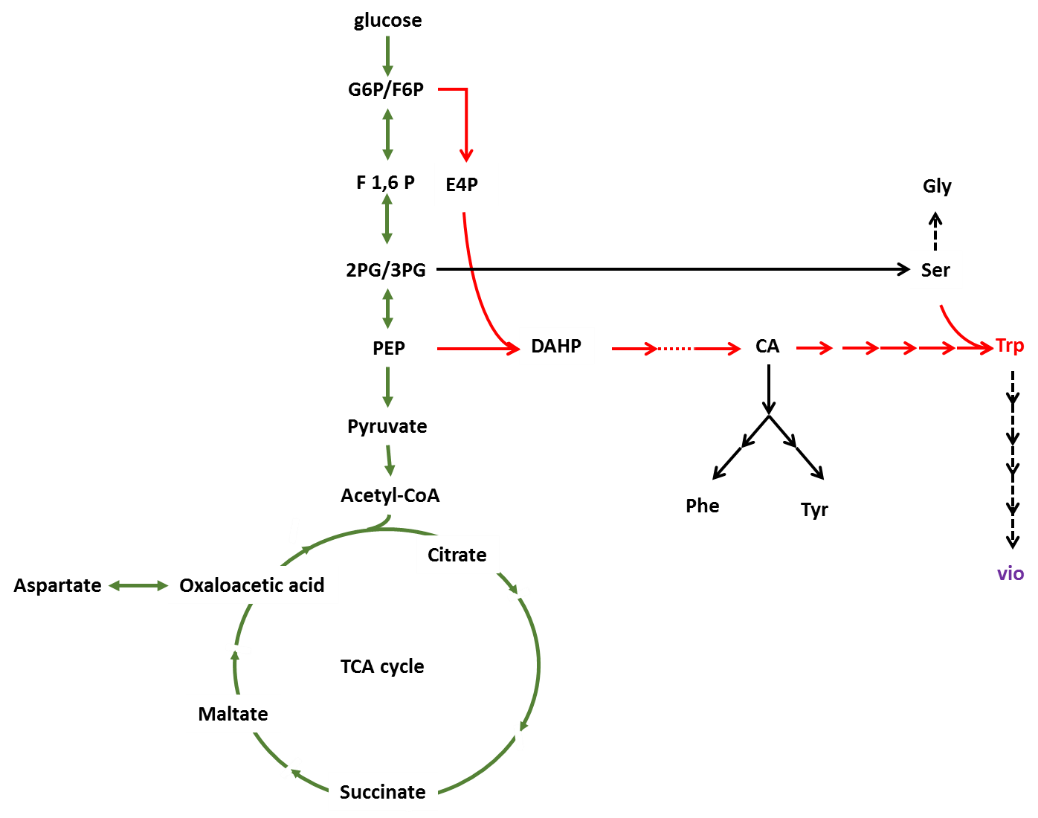


Figure S7B Pathway metabolic flux change of B2/pED+pVio compared with B1/pVio generated generated from Figure S7A, green and red mean relative slower and higher trend of pathway metabolic rate of B2/pED+pVio when compared with B1/pVio, respectively; Black means similar metabolic rate of these two strains; Dashed line means the metabolites in this pathway were not detected.

**Theoretical maximum yield of tryptophan toward glucose**.


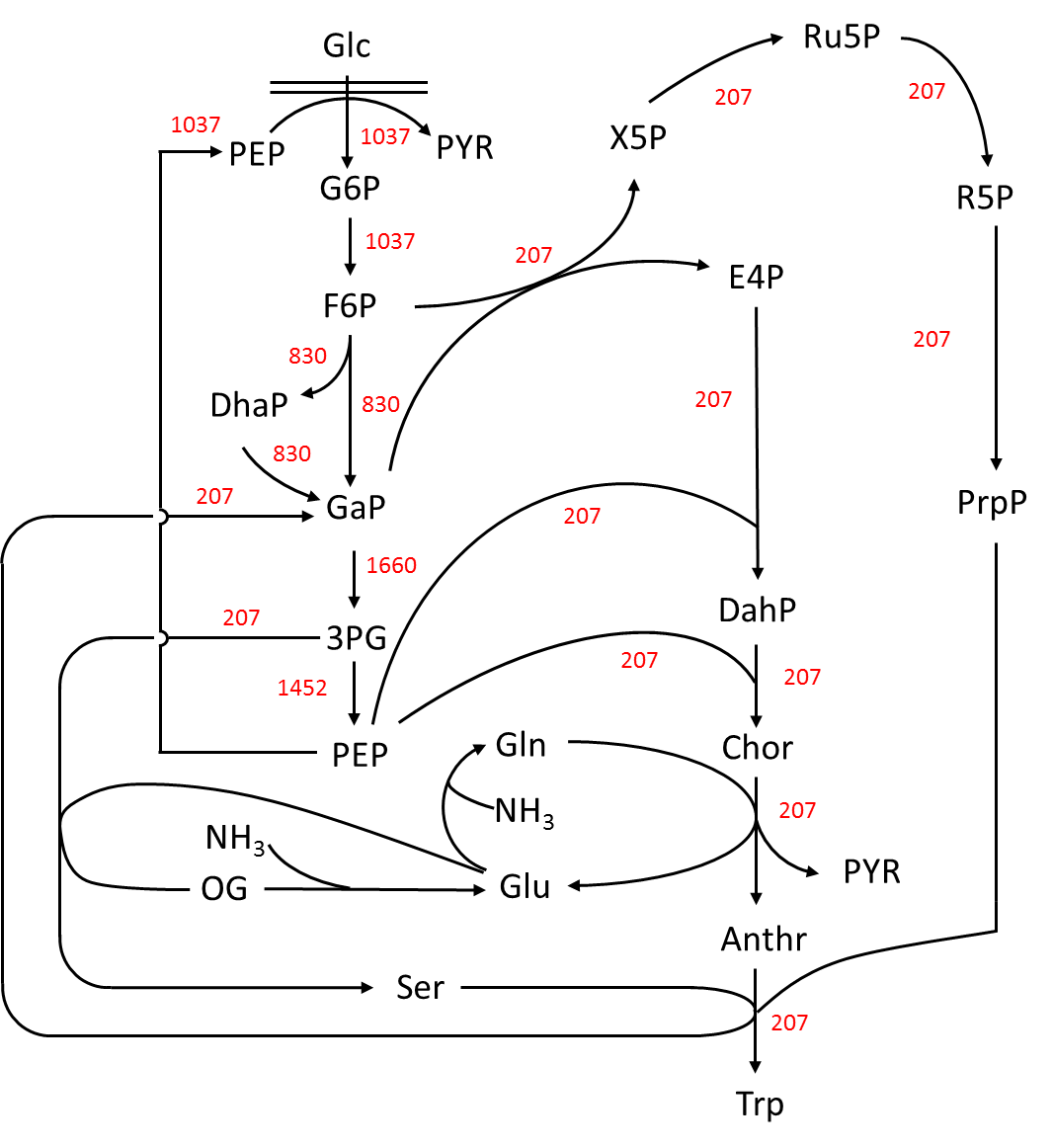


Figure S8A


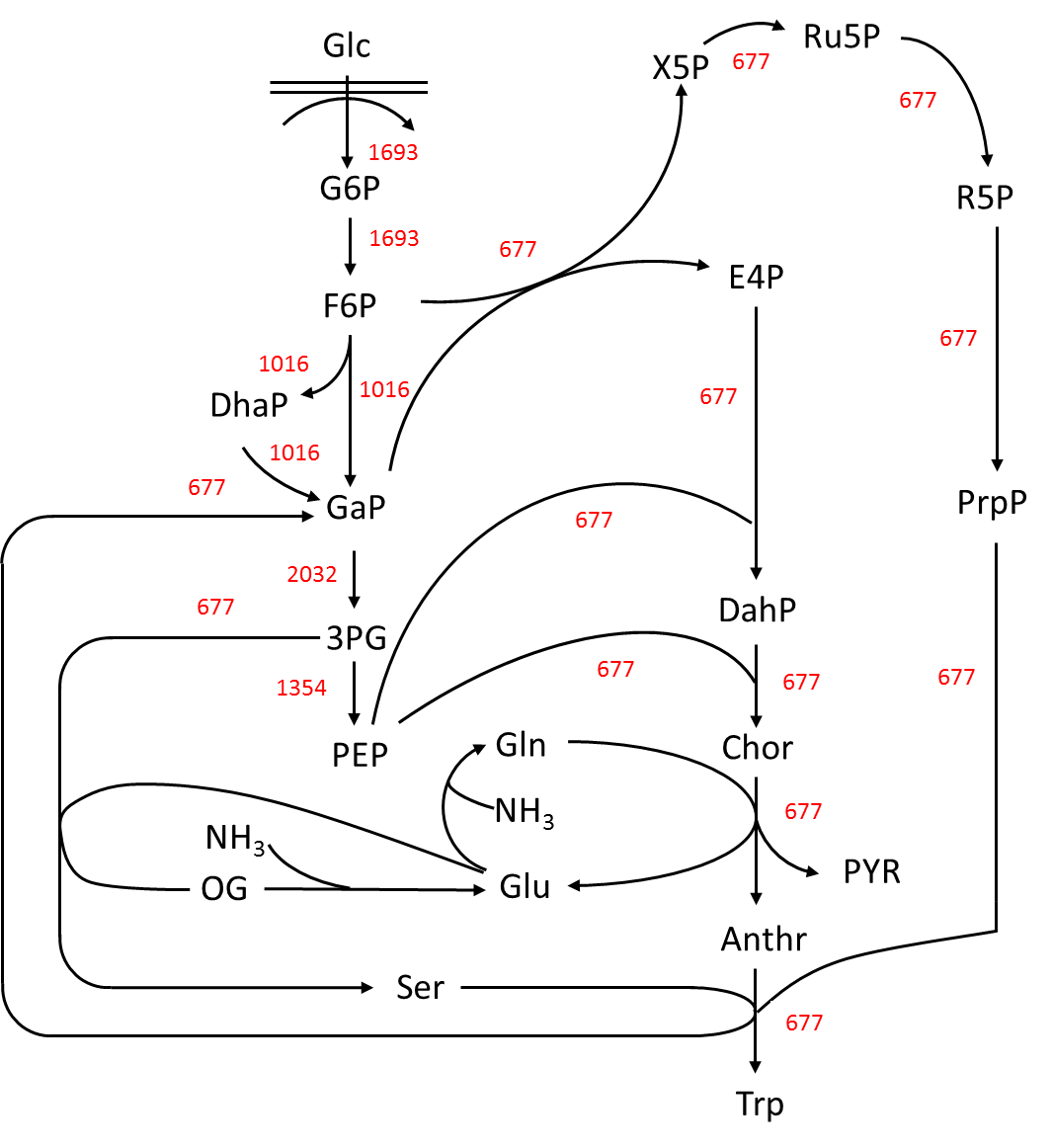


Figure S8B


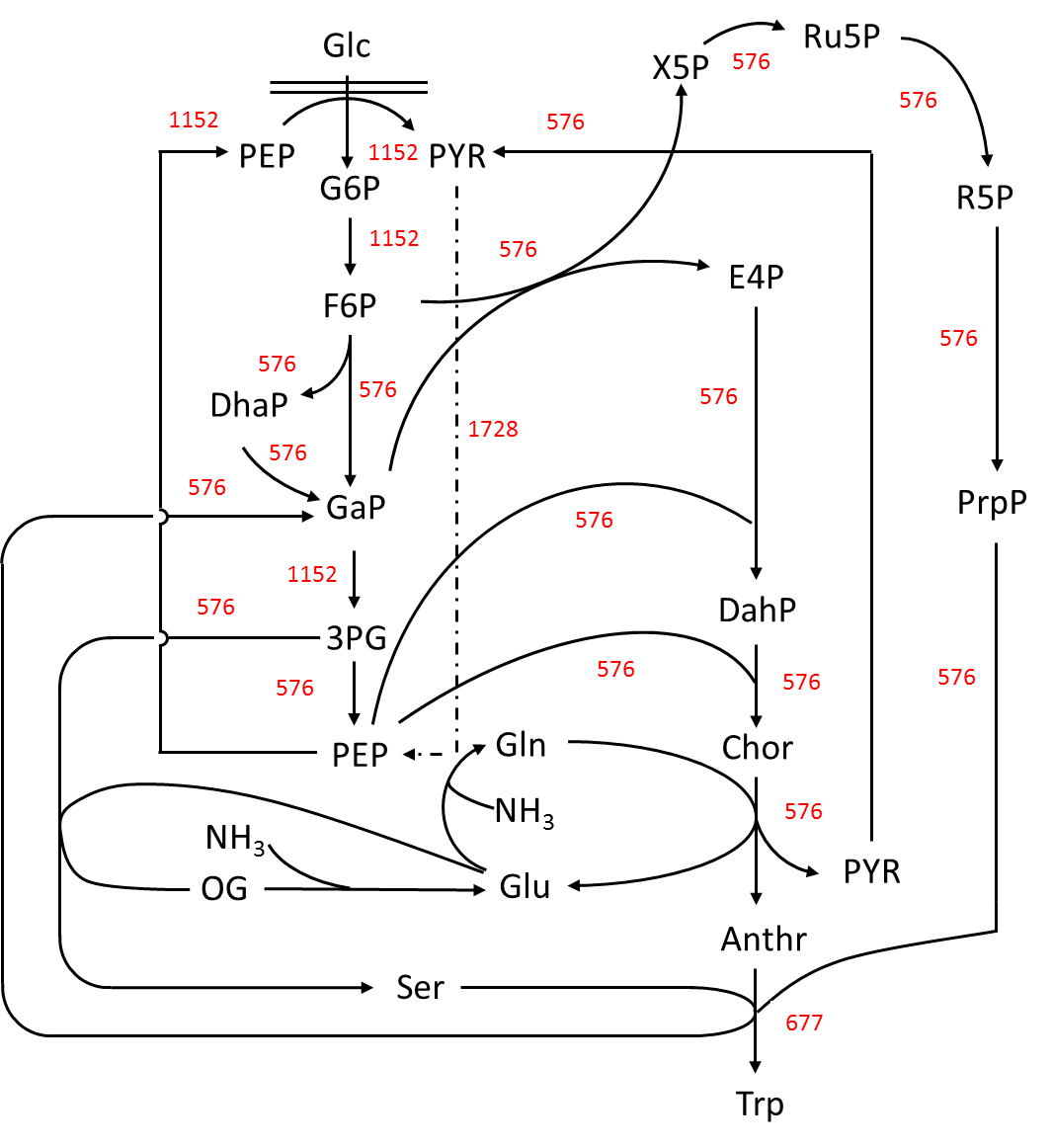


Figure S8C

Figure S8 Theoretical maximum yield of tryptophan toward glucose in absence of constant maintenance energy requirement. The numbers indicate the relative flux carried by the reactions. 8A, the theoretical maximum yield of tryptophan generated under common situation (0.20 molar yield); 8B, the theoretical maximum yield of tryptophan generated when the PTS is not taken into account (0.40 molar yield). 8C, the theoretical maximum yield of tryptophan generated when pyruvate is recycled back to PEP (0.59 molar yield). The metabolic figure was modified from Schuster S et. al [2], the yields were similar with previous published work under different situation [2,3,4,5]. Anthr, anthranilate; Chor, chorismate; DahP, 3-deoxy-arabinoheptulosonate-7-phosphate; DhaP, dihydroxyacetone phosphate; E4P, erythrose-4-phosphate; F6P, fructose-6-phosphate; GaP, glyceraldehyde-3-phosphate; Glc, glucose; Gln, glutamine, Glu, glutamate; G6P, glucose-6-phosphate; OG, 2-oxo-glutarate; PEP, phosphoenolpyruvate; 3PG, 3-phosphoglycerate; PrpP, phosphoribosylpyrophosphate; Pyr, pyruvate; R5P, ribose-5-phosphate; Ru5P, ribulose-5-phosphate; Ser, serine; Trp, tryptophan; X5P, xylulose-5-phosphate, PTS, phosphotransferase system.

[1] Klingenberg M, E P (1967) Means of terminating reactions: Academic Press.

[2] Schuster S, Dandekar T, Fell DA (1999) Detection of elementary flux modes in biochemical networks: a promising tool for pathway analysis and metabolic engineering. Trends Biotechnol 17: 53-60.

[3] Frost J, Lievense J (1994) Prospects for biocatalytic synthesis of aromatics in the 21st century . New Journal of Chemistry 341-348.

[4] Patnaik R, Liao JC (1994) Engineering of Escherichia coli central metabolism for aromatic metabolite production with near theoretical yield. Appl Environ Microbiol 60: 3903-3908.

[5] Varma A, Boesch BW, Palsson BO (1993) Biochemical production capabilities of Escherichia coli. Biotechnol Bioeng 42: 59-73.
